# Overcoming differential tumor penetration of BRAF inhibitors using computationally guided combination therapy

**DOI:** 10.1126/sciadv.abl6339

**Published:** 2022-04-29

**Authors:** Thomas S. C. Ng, Huiyu Hu, Stefan Kronister, Chanseo Lee, Ran Li, Luca Gerosa, Sylwia A. Stopka, Danielle M. Burgenske, Ishaan Khurana, Michael S. Regan, Sreeram Vallabhaneni, Niharika Putta, Ella Scott, Dylan Matvey, Anita Giobbie-Hurder, Rainer H. Kohler, Jann N. Sarkaria, Sareh Parangi, Peter K. Sorger, Nathalie Y. R. Agar, Heather A. Jacene, Ryan J. Sullivan, Elizabeth Buchbinder, Hannes Mikula, Ralph Weissleder, Miles A. Miller

**Affiliations:** 1 Center for Systems Biology, Massachusetts General Hospital Research Institute, Boston, MA, USA.; 2 Department of Radiology, Massachusetts General Hospital and Harvard Medical School, Boston, MA, USA.; 3 Department of Surgery, Massachusetts General Hospital and Harvard Medical School, Boston, MA, USA.; 4 Department of General Surgery, Xiangya Hospital, Central South University, Changsha, China.; 5 Institute of Applied Synthetic Chemistry, Technische Universität Wien, Vienna, Austria.; 6 Laboratory of Systems Pharmacology, Department of Systems Biology, Harvard Medical School, Boston, MA, USA.; 7 Department of Neurosurgery, Brigham and Women’s Hospital and Harvard Medical School, Boston, MA, USA.; 8 Department of Radiology, Brigham and Women’s Hospital and Harvard Medical School, Boston, MA, USA.; 9 Department of Radiation Oncology, Mayo Clinic, Rochester, MN, USA.; 10 Division of Biostatistics, Department of Data Sciences, Dana-Farber Cancer Institute, Boston, MA, USA.; 11 Department of Systems Biology, Harvard Medical School, Boston, MA, USA.; 12 Department of Cancer Biology, Dana-Farber Cancer Institute, Harvard Medical School, Boston, MA, USA.; 13 Department of Medicine, Massachusetts General Hospital and Harvard Medical School, Boston, MA, USA.; 14 Department of Medicine, Dana-Farber Cancer Institute, Boston, MA, USA.

## Abstract

BRAF-targeted kinase inhibitors (KIs) are used to treat malignancies including BRAF-mutant non–small cell lung cancer, colorectal cancer, anaplastic thyroid cancer, and, most prominently, melanoma. However, KI selection criteria in patients remain unclear, as are pharmacokinetic/pharmacodynamic (PK/PD) mechanisms that may limit context-dependent efficacy and differentiate related drugs. To address this issue, we imaged mouse models of BRAF-mutant cancers, fluorescent KI tracers, and unlabeled drug to calibrate in silico spatial PK/PD models. Results indicated that drug lipophilicity, plasma clearance, faster target dissociation, and, in particular, high albumin binding could limit dabrafenib action in visceral metastases compared to other KIs. This correlated with retrospective clinical observations. Computational modeling identified a timed strategy for combining dabrafenib and encorafenib to better sustain BRAF inhibition, which showed enhanced efficacy in mice. This study thus offers principles of spatial drug action that may help guide drug development, KI selection, and combination.

## Teaser

Image-guided computational pharmacology identified strategies to overcome poor tumor penetration of BRAF-targeted therapies.

## INTRODUCTION

Targeted kinase inhibitors (KIs) have been central to personalized medicine in oncology and are often prescribed on the basis of the presence of specific oncogenic mutations. However, their efficacy can be unpredictable in individual patients and must depend on factors beyond mere target expression or mutation. Inhibitors of mutant *BRAF* (v-Raf murine sarcoma viral oncogene homolog B) represent a key example of targeted therapy used in a genetically defined patient population: Malignant melanoma carrying an activating *V600* *BRAF* mutation exhibits robust initial responses to treatment with BRAF inhibitors (BRAFis) in combination with inhibitors of the mitogen-activated protein kinase (MAPK) kinase (MEK) kinase (MEKis), with greater than 50% objective response rate (ORR) in pivotal trials (*1*). Nonetheless, patients with melanoma often fail to respond to BRAFi/MEKi despite harboring V600 BRAF mutations, and in some cases, responses have been noted to BRAFi monotherapy despite progression on a prior course of treatment with a different BRAFi (*2*). This raises the question of how different inhibitors against the same targets may show distinct clinical activity on a patient-by-patient basis.

BRAFi efficacy has been reported in basket trials across diverse nonmelanoma BRAF-mutant cancers, although overall response rates are generally lower than as observed in melanoma (*3*). In metastatic colorectal cancer (mCRC), the ORR to BRAFi and BRAFi/MEKi combinations is much lower, <15% in some trials (*1*), and well-characterized BRAFi resistance mechanisms in mCRC include bypass signaling via epidermal growth factor receptor (EGFR) (*1*). However, combined inhibition in mCRC using dabrafenib (targeting BRAF), trametinib (targeting MEK1/2), and panitumumab (targeting EGFR) has exhibited mixed effectiveness, suggesting additional mechanisms of drug resistance (*4*). Intriguingly, second-generation BRAFi encorafenib outperforms first-generation vemurafenib in some contexts when combined with the MEKi binimetinib (*5*) and shows efficacy in combination with the EGFR-targeted antibody cetuximab to treat mCRC (*6*). Encorafenib is currently the only BRAFi shown to extend survival in mCRC, has received U.S. Food and Drug Administration (FDA) approval in refractory mCRC, and is undergoing trials in the mCRC frontline setting (all in combination with cetuximab). The success of encorafenib in mCRC compared to other BRAFi again raises the question of how different inhibitors against the same target may exhibit distinct clinical activities.

Understanding why related drugs behave distinctly in patients is crucial for (i) identifying the best drug to treat individual patients and (ii) guiding future drug development and combination regimens. Despite the abundance of documented routes to KI resistance, most studies implicitly presume that the primary factors are cell intrinsic and that KI exposure is adequate for good target coverage, in part based on known drug concentrations in circulation rather than in tumor tissue. In other words, past investigations have largely focused on adaptive pharmacodynamic (PD) rather than pharmacokinetic (PK) mechanisms of response and resistance (*7*).

Dosing and PK affect KI activity in complex ways (*8*), and relating serum drug concentrations to in situ drug exposure and subsequent tumor responses remains challenging. Biodistribution barriers for biologics, nanotherapies, and infused cytotoxics are widely appreciated (*9*, *10*), but barriers affecting orally administered small-molecule drugs have received less attention despite mass spectrometry studies highlighting variable KI delivery (*11*–*13*). Active drug transport can restrict drug accumulation in tumors, particularly with respect to the blood-brain barrier (BBB) for intracranial lesions (*14*); vemurafenib, dabrafenib, and encorafenib are all substrates of multidrug efflux transporters ABCB1 (MDR1/P-glycoprotein) and ABCG2 (BCRP) (*15*–*19*). Drug delivery barriers cannot simply be overcome by increasing dose for all patients: Dose-limiting toxicities of oral KI can be substantial. In pivotal trials, 67% of patients receiving BRAFi/MEKi combination dabrafenib/trametinib (D/T) experienced an adverse reaction leading to dose interruption (*20*). The balance between KI action in tumors and off-target tissues is therefore a concern, but the underlying mechanisms are unclear. This study thus presents a multipronged approach to address the question: To what extent does quantitative spatial PK/PD in the tumor microenvironment influence the efficacy of clinical BRAFi, and how might this information guide treatment strategies?

## RESULTS

### Clinical responses in BRAFi/MEKi-pretreated patients

**Fig. 1.**
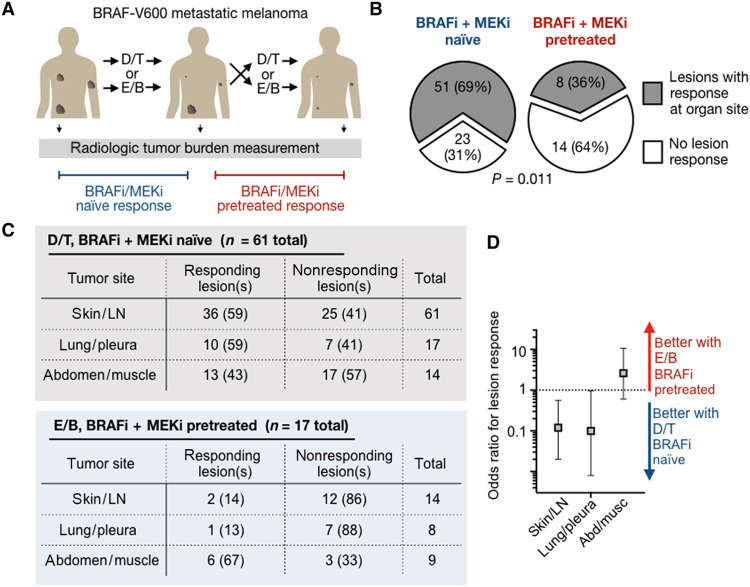
Patients exhibit distinct responses to BRAFi + MEKi following prior KI treatment. (**A**) Responses of tumor lesions to D/T or E/B were retrospectively analyzed across 81 pretreated or naïve patients. (**B**) Individual tumor responses were compared between pretreated and naïve cohorts (number of patients and Fisher’s exact test *P* value shown). (**C**) Responses of individual tumor lesions to D/T in BRAFi/MEKi-naïve patients or E/B in BRAFi/MEKi-pretreated patients were retrospectively analyzed by radiologic imaging. Lesion responses were binned according to organ site in patients with metastatic melanoma, reported as the number of organ sites showing lesion response and corresponding percentages in parentheses. (**D**) Odds ratios (means ± 95% CI) are shown corresponding to data in (C).

Because of shared drug targets and possibly shared resistance mechanisms, it is often hypothesized that BRAFi/MEKi pretreatment decreases responses to a subsequent round of therapy involving different BRAFi/MEKi as compared to responses in BRAFi/MEKi-naïve patients. Nonetheless, in the clinical setting, patients with BRAF-V600 melanoma often switch to a different BRAFi/MEKi combination, such as D/T or encorafenib/binimetinib (E/B), after progressing or experiencing toxicity with a prior BRAFi/MEKi treatment course, either D/T, E/B, or vemurafenib/cobimetinib. The frequency of such shifts presents an opportunity to quantify the degree of equivalence and cross-resistance to different BRAFi/MEKi combinations in patients. To examine this effect, individual tumor lesions from 81 patients with metastatic melanoma, receiving either D/T or E/B, under either BRAFi/MEKi-naïve or BRAFi/MEKi-pretreated conditions, were retrospectively analyzed (Fig. 1A). Most naïve patients received D/T, and most pretreated received E/B after D/T (fig. S2A).

As hypothesized, fewer BRAFi/MEKi-pretreated patients showed lesions with treatment response at a given organ site [as measured radiologically using metrics related to, but distinct from, RECIST (Response Evaluation Criteria in Solid Tumors); see Materials and Methods] compared to naïve patients (Fig. 1B and fig. S2B). This trend is consistent with past prospective trials comparing encorafenib or E/B radiologic response, as measured by RECIST criteria, in 127 combined BRAFi monotherapy–naïve versus BRAFi monotherapy–pretreated patients (table S1). However, pretreated patients in our analysis had also received prior MEKi, suggesting that responsiveness in the pretreated cohort is similar when MEKi is used throughout. Toxicity was a motivation for switching drug treatment in most cases, and it is possible that early switching due to toxicity may influence response rates during the second BRAFi/MEKi treatment course. Nonetheless, response to the second course of BRAFi/MEKi was observed in patients who had undergone prior BRAFi/MEKi treatment over a broad range of prior treatment durations (fig. S2C). These findings raise the possibility that different tumors may exhibit distinct sensitivity to different BRAFi/MEKi combinations on a lesion-by-lesion basis.

### Clinical BRAFi efficacy profiles suggest anatomic context dependency

Are E/B responses in D/T-pretreated patients a consequence of overall greater E/B efficacy? D/T and E/B have not been directly compared in randomized trials, but indirect comparison suggests that E/B fails to exhibit grossly superior clinical efficacy over D/T in melanoma (fig. S2D). We therefore hypothesized that potential differences between the drugs are context dependent. To study such possible differences, we analyzed whether melanoma lesion response depended on anatomical location (Fig. 1C). We used log-linear analysis to test whether response patterns at anatomical sites depended on the treatment (see the Supplementary Materials for details and rationale; fig. S2E). The analysis revealed two- and three-way interaction terms (fig. S2E): Responses depended on tumor site, tumor site occurrences were different across treatments, and responses depended on the treatment. Odds ratios showed a trend of skin and lymph node lesions responding more to D/T in BRAFi-naïve patients, compared to abdominal and muscle lesions (Fig. 1D). Together, these data suggest that D/T and E/B may exhibit distinct patterns of efficacy depending on the anatomical context of individual lesions.

### Systematic comparison of in vitro cytotoxicity

Are the clinical data results due to substantial differences in the in vitro on-target activities between different BRAFi and MEKi? In a past report, superior efficacy of encorafenib compared to dabrafenib was found using an in vitro cytotoxicity assay measured across a panel of BRAF-mutant cancer cell lines (*2*). However, new subgroup analysis of that data (*2*) revealed superiority only in highly sensitive cells, which may not be clinically relevant in the BRAFi/MEKi-pretreated setting examined here (fig. S3A). For a broader in vitro analysis, we queried the Broad Repurposing Library to analyze the magnitude of cell killing in experiments based on eight-point dose-response treatments across 388 cancer cell lines derived from diverse cancer types, including malignant melanoma, colorectal carcinoma, ovarian cancer, lung cancer, and others (fig. S3) (*21*). Encorafenib and dabrafenib responses correlated with each other (*R*^2^ = 0.49) and with BRAF mutation status of the individual cell lines (fig. S3B), but no consistent BRAF-dependent differences in cytotoxicity between dabrafenib and encorafenib or between the MEKi binimetinib and trametinib were noted, including within select cancer types (fig. S3, B to F). These results suggest that neither D/T nor E/B is broadly more potent on-target compared to the other with respect to in vitro activity.

### Companion dabrafenib imaging reveals heterogeneous in situ dose response

**Fig. 2.**
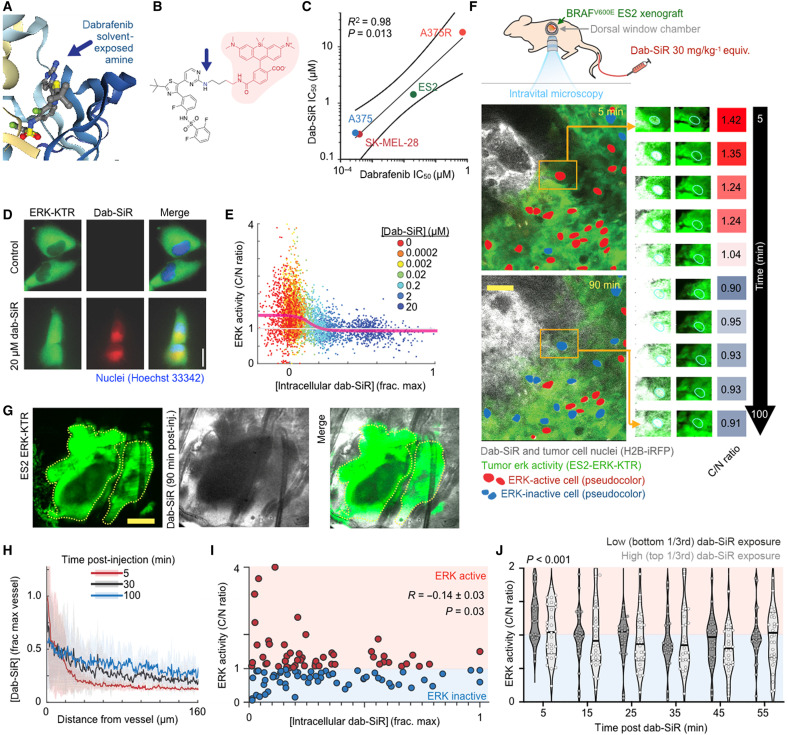
Companion dabrafenib imaging reveals heterogeneous single-cell PK/PD. (**A** and **B**) Crystal structure of dabrafenib bound to BRAF^V600E^ (A) (Protein Data Bank: 5CSW) and corresponding design of the near-infrared companion imaging drug, dab-SiR (B). (**C**) Dabrafenib and dab-SiR were compared across BRAF^V600E^ cell lines by 72-hour cytotoxicity (Pearson’s correlation and two-tailed *t* test reported; *n* = 2 reps). (**D** and **E**) Representative imaging of ES2-ERK-KTR cells treated ± dab-SiR for 2 hours (D) and corresponding cytoplasm-to-nucleus (C/N ratio) quantification (E) (*n* > 30 cells per condition); line denotes moving average of single-cell data. (**F** and **G**) Intravital microscopy of ES2 xenograft response to dab-SiR (30 mg/kg) using female nu/nu dorsal window chamber model at ×20 (F) (scale bar, 100 μm) and ×2 (G) (scale bar, 1 mm) magnification. Inset highlights single-cell response and corresponding quantification. (**H** to **J**) Drug concentration profile (H), ERK activity (I), and response after binning by drug exposure (J) were quantified from data as in (F). Data are means ± SE across three tumors and 90 cells. Two-way ANOVA (J) (*n* = 60 total cells) was used.

The apparent inability of in vitro cytotoxicity data (a form of PD) to explain distinct context-dependent patterns of clinical BRAFi/MEKi response motivated us to test the hypothesis that biodistribution or PK might play a role. We focused particularly on dabrafenib, since it exhibits high plasma protein binding, relatively rapid systemic clearance, and high lipophilicity compared to other relevant KIs. To relate drug delivery and action, a fluorescent companion imaging drug, dabrafenib silicon rhodamine (dab-SiR), was synthesized using the dye SiR-carboxyl (λ_ex/_λ_em_ = 652 nm/674 nm; Fig. 2, A and B) (*22*). Companion imaging drugs exhibit altered physicochemical properties as compared to the clinical compound, but they have unique advantages when it comes to studying PK (*23*). As anticipated, dab-SiR exhibited less efficacious biochemical median inhibitory concentration (IC_50_; 50 nM; fig. S4A) and reduced cytotoxicity (Fig. 2C) as compared to dabrafenib itself. Nonetheless, dab-SiR activity correlated with dabrafenib activity in a cytotoxicity assay across BRAF-mutant cancer cell lines (*R*^2^ = 0.98; Fig. 2C), including melanoma lines SK-MEL-28 and A375, the dabrafenib-resistant derivative A375R, and the ovarian clear cell carcinoma cell line ES2 as a model nonmelanoma BRAF-mutant cell line that is suitable for in vivo microscopy studies. These data thus suggest that other key properties of the labeled and parent drug are similar.

To compare local drug concentration to downstream effects on MAPK activity, dab-SiR was coimaged with an extracellular signal–regulated kinase kinase translocation reporter (ERK-KTR) (*24*). In this reporter, a fluorescent protein is fused to a synthetic ERK substrate whose phosphorylation causes the reporter to translocate from the nucleus to the cytoplasm. In vitro, ES2 *BRAF^V600E^* ovarian cancer ERK-KTR was found in the cytoplasm, indicating ERK activity, but the reporter translocated to the nucleus upon dab-SiR treatment (Fig. 2D), showing that BRAFi concentration can be correlated with activity at the single-cell level (Fig. 2E).

To relate BRAFi delivery with activity in the tumor microenvironment, ES2-ERK-KTR tumors were imaged by in vivo confocal (intravital) microscopy following dab-SiR. Briefly, xenograft tumors were implanted subcutaneously within dorsal window chambers of female nu/nu mice and imaged ~2 weeks later on a heated stage, following intravenous dab-SiR (Fig. 2, F and G). Histone 2B fused to near-infrared iRFP (H2B-iRFP) distinguished cell nuclei, which was differentiated from dab-SiR given the cytoplasmic localization of the latter. Although small-molecule drugs often extravasate rapidly into xenograft tumor tissue (*23*), penetration of dab-SiR was less extensive, and 30 min after drug injection, ERK-KTR activity was affected only in cells proximate to tumor vessels (<200-μm distance; Fig. 2, F to J). Even among cells in well-vascularized regions, those exposed to the highest dab-SiR concentration exhibited a slight reduction in ERK activity as compared to cells exposed to the lowest concentrations (Fig. 2, I and J). As a negative control, we showed that a coexpressed c-Jun N-terminal kinase (JNK)–KTR reporter was not inhibited by dab-SiR exposure (fig. S4, B and C), consistent with pathway-selective effects. Overall, these data reveal a heterogeneous initial signaling response to dab-SiR that correlates with uneven penetration of the drug from vessels into tumor tissue.

### Dab-SiR penetrates poorly into visceral metastases

**Fig. 3.**
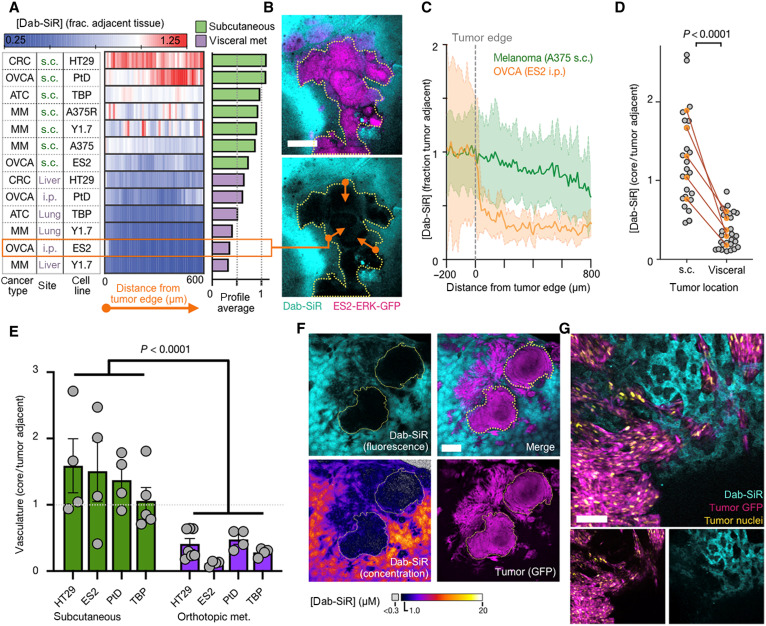
Dab-SiR penetration into solid tumors correlates with anatomical context and vascularization. (**A** to **D**) Mean radial line profiles quantify dab-SiR concentration as a function of distance from the tumor edge (A) (*n* ≥ 2 tumors per model), with representative omentum metastasis from ES2 (B) (scale bar, 100 μm) and individual line profiles (C) (thick line and shading denote means ± SE). Orange arrows illustrate radial profiles (multiple averaged per tumor). (D) Using models as in (A), dab-SiR was quantified in tumor center regions versus adjacent tissue, shown as individual tumor measurements (gray) and average values for matched subcutaneous (s.c.) and visceral metastasis models (two-tailed *t* test). i.p., intraperitoneally. (**E**) Corresponding to images as in (A), lectin was quantified in tumor center regions versus adjacent tissue, shown as individual tumor measurements (gray) and average values for matched subcutaneous and visceral metastasis models (*P* < 0.001, two-way ANOVA; *n* = 36 total tumors). (**F** and **G**) Confocal microscopy of dab-SiR in YUMMER1.7 (Y1.7) melanoma tumors in the liver at ×2 (F) (scale bar, 1 mm) and ×20 (G) (scale bar, 100 μm) magnification.

We next used dab-SiR imaging to test the generalizability of our clinical observations in mouse models of disseminated cancer. Using a panel of human, immunocompetent genetically engineered mouse, and patient-derived tumor models, dab-SiR penetration was assessed across BRAF-mutant tumors of melanoma, anaplastic thyroid cancer, ovarian cancer, and mCRC. Intravenous, intrasplenic, or intraperitoneal inoculation formed tumors in the lung, liver, or peritoneal cavity (omentum, ovary, and liver), respectively. Most tumors showed lower dab-SiR inside compared to outside tumors (Fig. 3A), and penetration was worse in metastases (Fig. 3, B to D, and fig. S5), despite the absence of consistent differences in tumor size (fig. S6). Average values across radial profiles (Fig. 3A) or over areas of ~0.25 mm^2^ within tumor cores (Fig. 3D) showed instances of >90% lower dab-SiR concentration compared to levels in adjacent tissue. Lectin quantified tumor vasculature, revealing that metastases were less functionally perfused than subcutaneous tumors (Fig. 3E and fig. S7).

Especially poor dab-SiR penetration was found in the orthotopic model of liver metastasis using genetically engineered melanoma cells (Fig. 3A). Dabrafenib is metabolized in the liver (*25*), and dab-SiR imaging showed hepatocyte accumulation (Fig. 3, F and G). Despite tumor invasion into adjacent sinusoid, little dab-SiR was found in tumor cells (Fig. 3G). Confocal imaging was compared to a standard ladder of dab-SiR to infer absolute concentrations, revealing tumor-averaged concentration of 0.8 μM, with >80% of tumor region showing <1 μM dab-SiR fluorescence (note that dab-SiR was administered at a molar equivalent of 10 mg/kg of dabrafenib in this experiment). These imaging data thus show >10-fold variation in dab-SiR concentration and can be quantified to guide subsequent computational modeling.

### Modeling in vivo mechanisms mediating BRAFi delivery

**Fig. 4.**
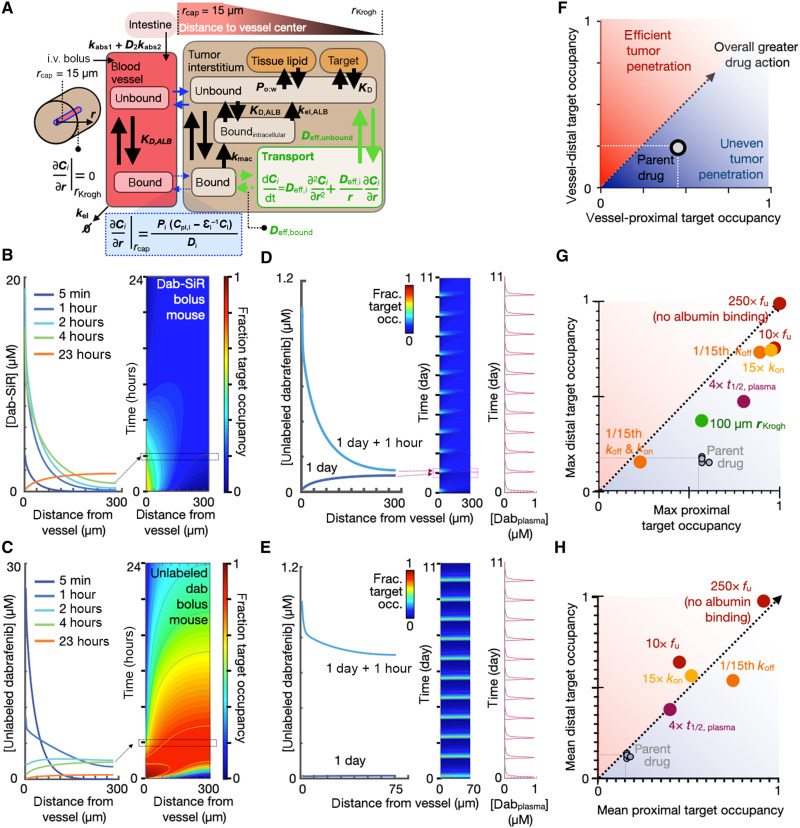
Multicompartmental kinetic modeling identifies albumin binding as an important factor for BRAFi tumor penetration. (**A**) Computational model schematic (tables S2 to S4 contain full equations and parameters). (**B** and **C**) Tumor concentration (left) and drug-target occupancy (right) of dab-SiR (B) and unlabeled parent dabrafenib (C) as a function of distance from tumor capillary and over time, modeled as a bolus [30 mg/kg, intravenously (i.v.)] in mice at *t* = 0. (**D** and **E**) Dabrafenib drug penetration over time and space, modeled with daily oral administration (30 mg/kg) in mice. Peak (+1 hour) and trough plasma concentrations at 1 and 5 days after treatment initiation are depicted, for poorly vascularized (D) (*r*_Krogh_ = 300 μm) and well-vascularized (E) (*r*_Krogh_ = 70 μm) tumors. (**F** to **H**) Parameter sensitivity analysis identifies factors affecting drug action for maximal (G) and mean (H) dabrafenib target occupancy, in cells nearest (*x* axis) or furthest (*y* axis) from vessels. Model parameters were adjusted as indicated, and results were compared to the model for parent dabrafenib depicted in (C).

We developed a simplified multicompartment model of spatial PK and drug-target binding to interpret the imaging observations from a quantitative and systematic perspective (Fig. 4A). A Krogh cylinder geometry was used to model drug penetration from a vessel into 50% of the tumor intercapillary distance, and free drug equilibrium between intracellular and extracellular compartments was assumed. Modeling was based on parameters derived from imaging data, measured in vitro, or already reported, including circulating half-life, lipophilicity, plasma protein binding, tumor vascularity, on-target (BRAF) association and dissociation, and others; in some cases, parameters were estimated from model compounds (tables S2 to S4; details in the Supplementary Materials). This approach allowed us to adjust model parameters to estimate drug behaviors that cannot be directly measured experimentally.

We first applied this model to simulate intravenously administered dab-SiR in poorly vascularized tumor tissue with a maximum intercapillary radius of 300 μm. This model matched imaging data in revealing a spatial gradient in drug concentration, target binding, and occupancy across early time points (≤4 hours) as a function of distance from blood vessels (Fig. 4B). By 24 hours, dab-SiR target occupancy is modeled as more homogeneously low after most of the drug has cleared circulation.

To better understand how dab-SiR imaging relates to the behavior of unlabeled dabrafenib, we next recalibrated the model to match known parameters of the latter, which we measured as exhibiting less lipophilicity and lower biochemical IC_50_ value than dab-SiR. We simulated behavior following intravenous injection (Fig. 4C) and found that a spatial gradient in target occupancy persists as a function of distance from blood vessels, with higher drug-target binding compared to the less potent dab-SiR. At the time of dab-SiR imaging 4 hours after injection, concentrations of parent dabrafenib were estimated to be roughly 0.5× to 2× the concentration of dab-SiR within the tumor core, depending on distance to vasculature. Thus, dab-SiR imaging of the melanoma liver metastasis model (Fig. 3, F and G) suggests that intratumoral variability in drug exposure is within ranges likely to affect cellular response (fig. S8): Cultured melanoma cells of this model show a cellular dabrafenib IC_50_ value of 0.3 μM [0.1 to 0.8 μM, 95% confidence interval (CI)] in a cytotoxicity assay. Notably, dabrafenib binding affinity to wild-type BRAF is similar between human and rodent, and we therefore did not adjust target-drug binding rates based on species in the modeling (*26*).

Since BRAFis are administered orally in preclinical efficacy studies (*27*) and in patients (*28*), we simulated daily oral dosing of parent dabrafenib in mice (Fig. 4D). Results showed heterogeneous target occupancy that was lower overall compared to intravenous administration, which is expected given the lower known plasma concentrations following oral treatment (Fig. 4D). Since well-vascularized tissues in humans have an intercapillary distance of ≤100 μm (*29*), we repeated simulations at this scale and observed spatially homogeneous drug-target binding (Fig. 4E). Thus, PK/PD modeling indicates that dabrafenib penetration depends strongly on vascularization, which correlates with the confocal microscopy data showing poor dab-SiR uptake in tumors with low functional vasculature (Fig. 3E).

To more systematically understand how individual PK/PD factors contribute to overall dabrafenib penetration and action, we artificially tuned parameters one by one, simulated intravenous dabrafenib as in Fig. 4C, and recorded target occupancy at the closest and furthest point to vasculature (Fig. 4F). Parameter alterations were made on the basis of clinically realistic adjustments (e.g., encorafenib exhibits 15× slower BRAF dissociation rate *k*_off_ compared to dabrafenib) or complete elimination of a process (e.g., the rate for direct cellular uptake of albumin, *k*_mac_ = 0). Among the parameters examined, drug penetration was most sensitive to albumin binding (Fig. 4, G and H, and table S5). In patients, the fraction unbound *f*_u_ for dabrafenib is 0.4% and is 35× higher for encorafenib. Increasing the fraction unbound by 10× leads to a predicted 1.7× increase in the maximum drug concentration reached in cells nearest to the vasculature, largely due to a greater availability of free drug to rapidly extravasate from vessels into tissue. Protein-bound drug transports much more slowly across vasculature by comparison. Increasing the fraction unbound drug by 10× has an even greater impact on drug concentrations furthest from the vasculature (>4-fold enhancement), since free drug is modeled as diffusing faster through tumor interstitium compared to protein-bound drug.

In our simplified model, once drug partitions into lipid, it does not interstitially transport or bind its target. Under these simplifications, simulations indicate the relative insensitivity of target occupancy to lipophilicity. In reality, drug-lipid partitioning effects can be complex. Nonetheless, simulations also show that increased lipophilicity leads to greater total accumulation that is more spatially heterogeneous, which affects imaging (table S5).

The dissociation constant *K*_d_ is defined by the ratio of *k*_off_/*k*_on_ (rates of drug-BRAF dissociation *k*_off_ and association *k*_on_), and modeling confirms that *K*_d_ is a main determinant of target inhibition. However, achieving lower *K*_d_ via slower *k*_off_ leads to more evenly sustained target binding in vessel-proximal cells (Fig. 4H). In comparison to the case with slower *k*_off_, achieving lower *K*_d_ via a faster *k*_on_ leads to more spatially and temporally variable, but greater peak, inhibition (Fig. 4G). Collectively, modeling indicated that circulating half-life, on-target binding rates, vascularization, and especially albumin binding combine to influence spatially dependent dabrafenib action.

### Comparing transport of encorafenib and dabrafenib

**Fig. 5.**
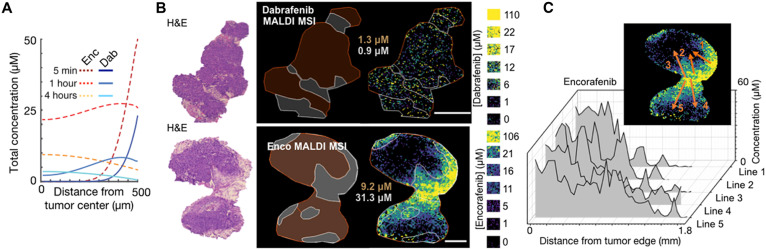
Encorafenib and dabrafenib exhibit distinct heterogeneous tumor penetration. (**A**) Simulation of parent dabrafenib or encorafenib, given by intravenous bolus in mice, and their penetration from a fully perfused margin into the avascular center of a 1-mm spherical tumor. (**B**) Representative mass spectrometry imaging (MALDI MSI) of unlabeled dabrafenib and encorafenib and corresponding standard tissue phantoms (calibration curves shown in fig. S9). Tissues were analyzed 4 hours after injection in the subcutaneous YUMMER1.7 melanoma model. Regions highlighting dense tumor (brown) versus stroma, as guided by hematoxylin and eosin (H&E), with corresponding mean drug concentrations are shown on the left (scale bars, 1 mm). Line profiles depicting drug concentration are shown in the inset and graphed in (**C**).

With evidence that albumin binding may hinder drug penetration, we hypothesized that parent (unlabeled) encorafenib may be less impeded by albumin and better accumulate in tumors compared to parent (unlabeled) dabrafenib. We used a computational model as described above, but with tumors instantiated as spherical avascular lesions within well-perfused tissue, and adjusted for correct dose and drug PK/PD properties. Tumor penetration and uptake were predicted to be more efficient with encorafenib (4 to 9 μM, depending on vascular proximity) compared to dabrafenib (0.5 to 4 μM) at 4 hours after injection (Fig. 5A). Matrix-assisted laser desorption/ionization mass spectrometry imaging (MALDI MSI) is an alternative way of measuring the spatial distributions of small molecules and drugs in tissue sections (*30*). In the same metastatic mouse model of melanoma as imaged with dab-SiR, MALDI MSI measured the penetration of dabrafenib and encorafenib into tumors, and concentrations of the latter were found sevenfold higher than those of the former (Fig. 5, B and C, and fig. S9). This difference roughly matches the modeling prediction in Fig. 5A and is opposite of what naïvely might have been expected since encorafenib dose was threefold lower than that of dabrafenib. Robust encorafenib MALDI MSI signal allowed us to examine spatial heterogeneity by plotting drug concentration along radial lines from stroma into the tumor, revealing even greater variability than predicted by the simplified computational model. Higher stromal vascularization and lipid content could potentially explain the high concentrations observed in the adjacent subcutaneous tissue in the MALDI MSI. Thus, MALDI MSI supports the hypothesis that KI tumor penetration may be inefficient.

To quantify the potential impact of differences in drug concentration observed by MALDI MSI, we requeried the Broad Repurposing Library used in fig. S3. Relative impact on proliferation/cytotoxicity across 60 BRAF-mutant cell lines was compared in response to either dabrafenib or encorafenib (fig. S10). For both drugs, increased cytotoxic effects were observed going from 0.6 to 2.5 μM, and these levels are within the range of variability observed in vivo (fig. S10, A and B). Encorafenib did not exhibit enhanced cytotoxic effects compared to dabrafenib when compared at equimolar concentrations, but encorafenib was more effective when compared to dabrafenib at the unequal doses within the range of values observed in vivo (fig. S10B). Twenty-four of 60 cell lines showed double the reduction in cell count with 10 μM encorafenib, compared to 0.6 μM dabrafenib. It is therefore likely that distinct local concentrations of encorafenib and dabrafenib achieved in tissue may lead to distinct tumor responses to the two drugs.

**Fig. 6.**
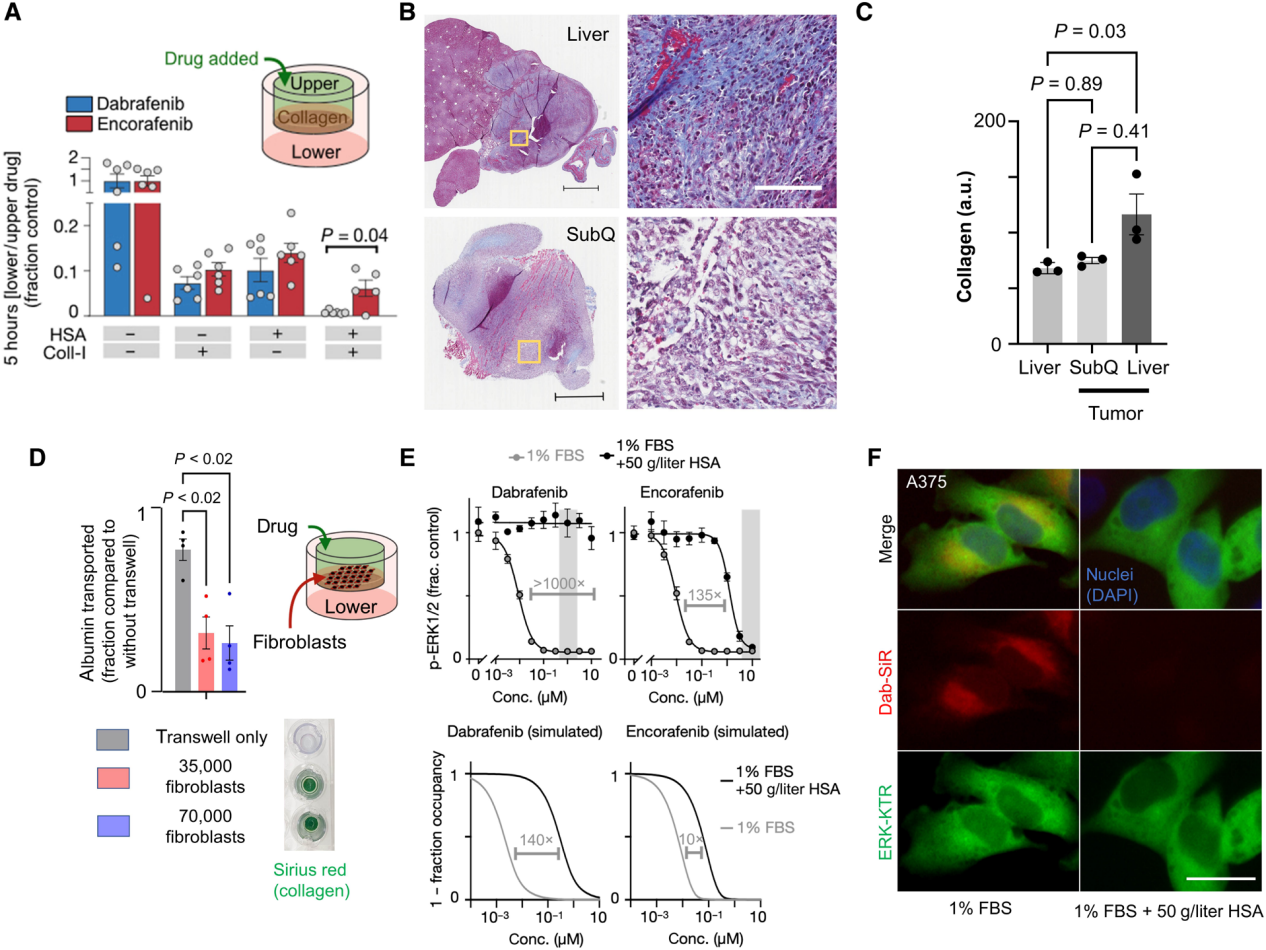
Albumin binding limits diffusion through collagen and cellular uptake. (**A**) Transwell measurement of unlabeled drug transport from upper to lower chamber by liquid chromatography–mass spectrometry (means ± SEM, two-tailed *t* test, *n* ≥ 5). (**B**) Representative visceral (top) and subcutaneous (bottom) YUMMER1.7 melanoma tumors stained for collagen using Masson trichrome (blue). Inset (yellow boxes) shown at the right. Scale bars, 500 and 100 μm (inset). (**C**) Collagen quantified from Masson trichrome in (B) (*n* = 3, means ± SE, Kruskal-Wallis test). (**D**) Transwell measurement as in (A) was used with activated fibroblasts rather than collagen gel. Alexa Fluor 647–albumin transport from top to bottom chamber was measured after 3 hours (means ± SE, two-way ANOVA corresponding to fig. S11). Transwell inserts were stained with Sirius red. (**E**) p-ERK1/2 immunofluorescence of A375 cells treated for 2 hours in the presence or absence of physiologic HSA concentrations (top, with observed in vivo drug concentration range shaded) and corresponding computational modeling predictions based on drug PK/PD properties (bottom). (**F**) Dab-SiR drug uptake in the presence of albumin for A375 cells (1 μM; incubated for 2 hour). a.u., arbitrary units; DAPI, 4′,6-diamidino-2-phenylindole.

We hypothesized that different albumin binding affinities between dabrafenib and encorafenib could contribute to differences in their observed tumor penetration. We used an in vitro transwell assay to test this hypothesis and found that drug transport was comparable between encorafenib and dabrafenib, except for when transwells were both separated by collagen and drug was premixed with HSA (human serum albumin): In this case, dabrafenib was transported significantly more poorly than encorafenib (Fig. 6A). This observation may have implications in vivo; diffuse collagen infiltration within visceral tumor stroma was noted compared to surrounding parenchyma and tended to be increased compared to subcutaneous lesions in a mouse melanoma model studied (Fig. 6, B and C); novel fibroblast and collagen-targeted clinical imaging agents have also shown significant uptake across multiple tumor types (*31*, *32*). As cancer-associated fibroblasts are implicated in stromal collagen modeling (*33*, *34*), we also tested the effect of activated fibroblasts on albumin transport using an in vitro transwell assay. This assay found that albumin transport was significantly impeded by the presence of collagen-secreting activated fibroblasts (Fig. 6D and fig. S11). Since encorafenib is known to bind less to plasma protein compared to dabrafenib, and free drug diffuses through collagen faster than protein-bound drug, these data suggest that albumin binding may have a greater impact on limiting the transport of dabrafenib through tumor stroma, compared to encorafenib.

In principle, albumin binding can also affect drug action in cell cultures where diffusion/convection is less important, since protein-bound drug is prevented from engaging in its target. To directly test whether albumin differentially affects KIs including dabrafenib and encorafenib, we treated A375 melanoma cells with a KI dose response for 2 hours in the presence or absence of physiologic levels of HSA and measured downstream p-ERK1/2 levels by immunofluorescence (Fig. 6E). In agreement with the computational model used above (but adapted for a well-mixed cell culture), both drugs were inhibited by HSA, and dabrafenib was more inhibited than encorafenib. Experimental data showed a greater magnitude of HSA effect overall than predicted by the model, potentially due to additional physicochemical (e.g., viscosity/diffusion), extracellular/intracellular partitioning, and biological signaling impacts of HSA in serum-deprived cells. Nonetheless, data are consistent with tighter binding of HSA with dabrafenib compared to encorafenib. Assessment of ERK-KTR activity, as well as attenuated cellular uptake of dab-SiR in the presence of albumin, also supports this assertion (Fig. 6F and fig. S12, A and B). Together, these data and PK/PD models illustrate how HSA binding can limit both interstitial transport and target engagement.

To understand how findings in mice and cell culture may translate to human patients, we recalibrated our computational models of encorafenib and dabrafenib to approximate clinical dosage and PK. Daily oral dosing of encorafenib resulted in more sustained drug-target binding, even far from vasculature. By contrast, dabrafenib penetration and target occupancy were indicated to be comparatively decreased, even when given twice daily as done clinically (fig. S10, C and D). Thus, concentration differences found in tumors may be significant in clinically relevant dosage schemes and may influence treatment efficacy, particularly in less vascularized tumors where drug penetration issues are exacerbated.

### Drug penetration into intracranial melanoma lesions

Several factors have been implicated as barriers for effective BRAFi delivery to intracranial lesions, including active drug efflux (*17*, *19*), and BBB integrity (*35*, *36*). Nonetheless, BRAFis are used to manage adult and pediatric BRAF-mutant metastatic and primary brain lesions (*37*–*39*). To understand how our observations in extracranial sites may also apply intracranially, we used two models of melanoma metastasis to the brain.

**Fig. 7.**
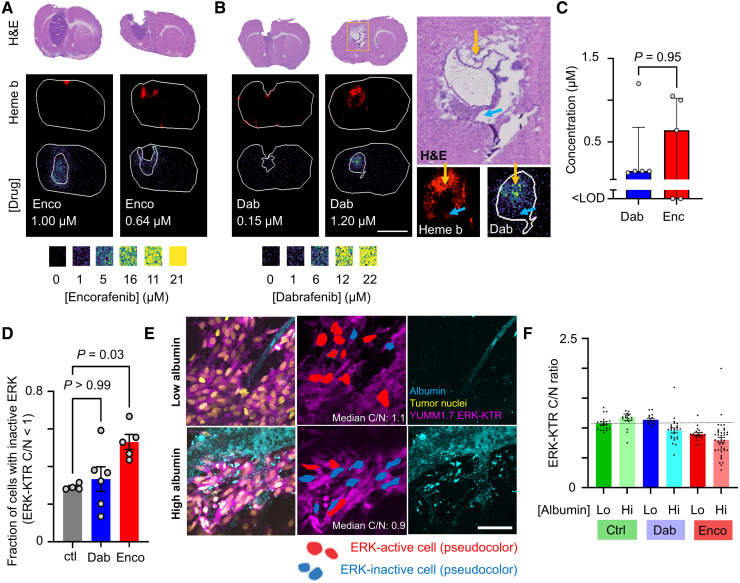
Heterogeneous BRAFi uptake and response in intracranial metastases. (**A** and **B**) Representative MALDI MSI of encorafenib (A) and dabrafenib (B) and corresponding quantification (C) in an intracranial PDX model of metastatic melanoma, assessed 4 hours after intravenous drug injection as in Fig. 5. Regions highlighting tumor, as guided by H&E, with corresponding mean drug concentrations are shown. Scale bar, 4 mm. Heme b marks vasculature and blood. (B) Magnified inset at the right highlights high (yellow arrow) and low (blue arrow) uptake. (**C**) Drug concentrations across intracranial lesions (median ± interquartile range, Mann-Whitney *U* test, *n* = 5). (**D**) Fraction of cells showing low ERK activity 4 hours after treatment in intracranial YUMM1.7 melanoma micrometastases (means ± SE, *n* ≥ 4 animals, Kruskal-Wallis test). (**E**) Corresponding to (D), representative encorafenib-treated micrometastases. Scale bar, 50 μm. (**F**) Corresponding to (D) and (E), ERK-KTR activity across tumor regions showing low or high albumin exposure (*N* ≥ 3 animals per condition across 359 total cells, means ± SE).

Mice with intracranially implanted melanoma patient-derived xenograft (PDX) were treated intravenously with either dabrafenib or encorafenib, and 4 hours later, brains were dissected and processed for MALDI MSI. This analysis revealed, on average, 4-fold less dabrafenib and 24-fold less encorafenib within brain lesions compared to levels seen in the extracranial melanoma allograft tumors (Fig. 5), at the same time of 4 hours after injection of drug. Nonetheless, higher drug in tumor than matched normal brain parenchyma and the presence of heme near or within tumors suggest altered vasculature and BBB breakdown. BRAFi tumor penetration was heterogeneous for both drugs, with 0.15 and 0.64 μM median values for dabrafenib and encorafenib, respectively (Fig. 7, A to C). Overall, these results indicate diminished and variable drug penetration in brain lesions that likely depends on BBB compromise.

For a single-cell–level understanding of heterogeneous intracranial BRAFi delivery, we used an immunocompetent model of brain metastasis based on intracardiac injection of genetically engineered melanoma cells expressing the fluorescent ERK-KTR reporter. As above, tumor-bearing mice were intravenously treated with BRAFi and brains were excised 4 hours later for imaging. Fluorescently labeled HSA was coinjected to report on BBB function and albumin-bound drug transport. Confocal microscopy quantified ERK pathway inhibition in micrometastatic brain lesions using the ERK-KTR reporter (Fig. 7D), showing pathway inhibition in response to encorafenib. In contrast, dabrafenib elicited mixed responses that did not consistently differ from the control-treated mice but induced a higher variance in ERK activity across the treated mice (*P* = 0.006, *F* test with Bonferroni correction). Tumor regions with high albumin extravasation showed greater pathway inhibition than regions with low albumin uptake when BRAFi was applied [*P* = 0.0003, two-way analysis of variance (ANOVA) interaction term describing combined effects of treatment and albumin levels on ERK-KTR activity; Fig. 7, E and F]. These findings suggest that heterogeneous BBB permeability and drug delivery could account for mixed cellular responses to treatment.

### Enhancing efficacy through model-guided BRAFi combination

**Fig. 8.**
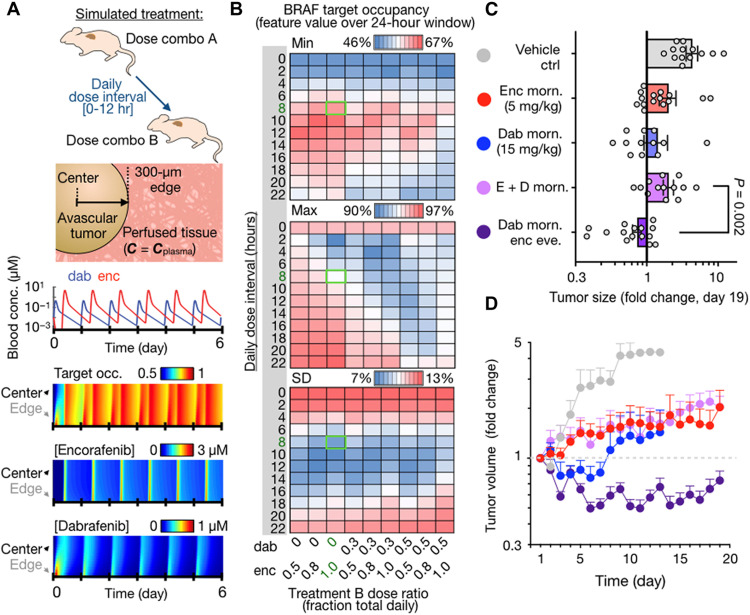
Combined dabrafenib and encorafenib treatment more effectively blocks tumor growth when dosing is staggered. (**A**) Top: Proposed dose combination strategy and computational tumor model. Middle: Simulated blood concentrations of total drug are shown for staggered treatment combination, spaced 8 hours apart. Bottom: Simulations of BRAF target occupancy and total drug concentrations as a function of distance from tumor edge, over the course of a 6-day treatment period for staggered dabrafenib and encorafenib. (**B**) Simulated heatmaps showing the fraction of BRAF that is bound by drug as a function of drug fractionation across the first and second daily doses (*x* axis) and daily dose interval (*y* axis). Green box highlights the selected dosing scheme for experiments and corresponds to simulations shown in (A). (**C** and **D**) Braf-mutant melanoma allograft growth in C57Bl/6 mice was monitored by caliper in response to BRAFi regimens, shown as individual tumor data at day 19 after treatment (C) and over time (D) with matched color labeling. Data are means ± SEM, across 69 total tumors in 19 mice (two-tailed Welch’s *t* test).

Given (i) the clinical observations that different BRAFi may exhibit distinct clinical activities on a patient-by-patient level and (ii) that dabrafenib and encorafenib exhibit distinct and suboptimal PK and tumor penetration behaviors, we next examined the potential benefit of combining the two BRAFis to achieve more sustained target inhibition. We first performed computational simulations to model BRAFi target occupancy under a variety of clinically realistic dosage schemes (Fig. 8A), at doses near the maximum tolerated dose we observed in mice bearing hepatic melanoma lesions (fig. S13A). Doses of dabrafenib and/or encorafenib were given in silico in the morning, and in some cases, a second distinct dose was given later in the day, and other model parameters matched prior analyses (Fig. 5A). BRAF target occupancy was then recorded as a function of time and distance from the vascularized tumor edge into its avascular tumor core. Simulations were performed over dose intervals ranging from 0 to 12 hours and across a range of fractionations such that a drug was given in the first daily dose or the second daily dose or split across both daily administrations. Minimum, maximum, and SD in BRAF target occupancy were then recorded over the last 24-hour window of the simulation (Fig. 8B). The model indicated that combining the two drugs would not be beneficial if they were given at the same time once daily. In contrast, improvement in sustained target inhibition was predicted if the drugs were given in a staggered manner (Fig. 8B). This observation applied to tumors with high or low vascularization. Administering dabrafenib in the morning and encorafenib in the evening was predicted to be more effective than the reverse, largely because encorafenib is longer-acting (via slow *k*_off_ rate) and better sustains BRAF inhibition in the 16-hour overnight dose interval compared to dabrafenib (Fig. 8B and fig. S13A).

On the basis of these simulations, we tested the longitudinal efficacy of dabrafenib and encorafenib combination therapy in genetically engineered Braf^V600E^-mutant YUMM1.7 allografts, using immunocompetent male C57Bl/6 mice. Upon subcutaneous tumor formation, subjects were treated with a subset of drug regimens that had been computationally simulated, and tumor volumes were monitored daily by caliper. In agreement with the modeling results, staggered treatment with dabrafenib in the morning and encorafenib in the evening was most effective at blocking tumor growth (Fig. 8, C and D). In contrast, administering both drugs simultaneously did not show a benefit over single-agent treatment. In addition to reducing average tumor growth over time across the cohort (Fig. 8, C and D), the staggered treatment also yielded more consistently controlled tumor growth. The coefficient of variation in tumor growth was highest in the dabrafenib single-agent treatment group and lowest in the staggered combination treatment group (fig. S13B). Variance across the individual tumors within each group was also significantly lower (*P* < 0.0001, *F* test). These results are consistent with a more sustained and spatially homogeneous target inhibition in the staggered treatment group and more variable drug action in the dabrafenib-only treatment group, which were the predictions from the computational model. Average body weight loss was <10% for all combination regimens (fig. S13C). Overall, this experiment indicates that dabrafenib and encorafenib can be combined to outperform single-agent treatment, when doses are appropriately timed.

## DISCUSSION

This report combines chemical biology tools with in vivo microscopy and clinical imaging to understand why some KIs may work better than others on a patient-by-patient and lesion-by-lesion basis, despite sharing common drug targets and treating tumors with similar genetic mutations. Populating in silico pharmacology models with imaging data ultimately revealed a potential role for combining multiple BRAFis together to achieve more sustained target inhibition. Across a panel of mouse and patient-derived tumor models, we found wide variation in the ability of KIs to penetrate tumor tissue, particularly in poorly vascularized metastatic sites within the liver and abdomen. In some cases, drug concentrations within the tumor were decreased by >90% compared to levels in adjacent nontumor tissue (Fig. 3D). Computational modeling based on experimental- and literature-derived parameters suggests that differential drug penetration is relevant in patients. Are such variations in drug concentration clinically significant? Anecdotally, in the Ph-I BRF112680 for dabrafenib, oral 200 to 400 mg/day exhibited 40% ORR when pooled across cohorts, compared to 90% in the high-dose 600 mg/day (300 mg, twice daily) cohort (*40*). Poor BRAFi penetration may also amplify dose-dependent pro-resistance signaling (*41*) and has implications for understanding the mechanisms of drug-drug interaction (e.g., synergy) for BRAFi/MEKi combinations (*42*), since the two drugs may distinctly accumulate in different tumor cell subsets and show differential tumor penetration.

BRAFi and BRAFi/MEKi combination therapies have shared toxicity profiles that appear to be class effects (*43*). However, D/T and E/B also exhibit distinct characteristic toxicities that create challenges for delivering full-dose therapy. Indirect comparisons of adverse event rates across pivotal D/T and E/B trials highlight these differences, indicating pyrexia as problematic for D/T and elevated AST (aspartate aminotransferase) as problematic for E/B (fig. S13D). As an alternative to combining different BRAFis, our computational model predicted that simply doubling the dose of encorafenib and administering twice daily would also be effective (fig. S13A). However, mice bearing hepatic melanoma lesions lost body weight when encorafenib dose was increased from 10 to 15 mg/kg, despite showing decreased tumor burden (fig. S13F), and therefore, we did not pursue this strategy. It is thus attractive to consider that nonoverlapping toxicities may be mitigated, while preserving or enhancing sustained target inhibition, by combining drugs together at lower individual doses. Future work should examine such toxicity implications, including when multiple BRAFis may be combined with MEKi or EGFR-targeted therapies.

Limitations of this work must be considered. Imaging sensitivity required trade-offs between signal-to-noise and steady-state biodistribution. Zero- to 4-hour post-injection was chosen as a compromise, with intravenous rather than oral administration to minimize variable uptake and potential fluorophore influence on bioavailability. PK/PD models were used here to clarify such effects. As with all such models, these represent a simplification of the underlying physiologic processes. Nonetheless, correspondence of our modeling results with experimental data highlights the utility of modeling to mechanistically interpret our findings. Subsequent studies may also directly analyze the metabolite distribution of fluorescent drug conjugates, since currently the confocal approach provides only a composite measurement. Drug metabolization and transport are interconnected processes; direct comparison between relevant metabolites of dabrafenib and encorafenib, along with their physical properties and relative contributions to overall drug activity, should be performed in future studies. Dabrafenib binding effects on albumin function in vivo should also be further considered (*44*). Other tumor microenvironmental factors, such as acidity, may also affect drug penetration through modulation of protein binding and lipophilicity (*45*). Recent pathological studies also suggest that histological growth patterns of visceral metastases, particularly melanoma and colorectal lesions, can affect their vascularity and treatment response (*46*–*48*). The poorly vascularized replacement pattern portended an especially poor prognosis (*49*). The retrospective data presented here did not have correlated histopathology collected, but collection of these data, either via biopsy or by noninvasive imaging assessment, for example, with albumin-binding (*50*, *51*) and fibroblast-targeting agents (*31*, *32*), should be considered in future prospective evaluations. Last, retrospective clinical data suggest context-dependent differences in activity between D/T and E/B and motivate randomized prospective comparisons between the two drug combinations, which would better control for possible confounders including toxicity, impacts of temporary breaks in treatment, and heterogeneous tumor genetics across metastases.

How generalizable are the findings to other drugs? Some drugs have been formulated to bind albumin, including nanoparticulate albumin-bound paclitaxel (nab-paclitaxel), which, in part, allows drug to accumulate more efficiently in RAS-mutant tumors by exploiting their macropinocytic appetite for albumin as a nutrient source (*52*). MAPK/ERK pathway inhibition may block macropinocytosis and tumor uptake of albumin, therefore reducing the potential advantages of albumin binding for drug delivery (*53*). Among FDA-approved drugs for oncology, 21 with ≥99% plasma protein binding are lipophilic (table S6) (*54*). Most are used for applications with perhaps less challenging PK barriers, including blood and skin cancers, local (e.g., topical) administration, antiangiogenics, or hormone modulation. Highly protein-bound and lipophilic drugs being tested for solid cancers, such as venetoclax and navitoclax (e.g., NCT01989585), may face tumor penetration challenges. Drug plasma protein binding can prolong drug circulation and be advantageous for drug delivery in many cases (*55*). However, in general, there remains a poor correlation between circulating half-life and plasma protein binding across diverse drug structures, and debate continues over the role of albumin binding in drug delivery (*56*, *57*). Here, we find that albumin binding can influence the spatial penetration of drug into solid tumors, particularly for dabrafenib, which exhibits relatively fast initial clearance kinetics (*t*_1/2initial_ < 2 hours) despite high albumin binding (*40*), and especially for poorly vascularized and fibrotic lesions, such as seen in liver metastases. Notably, clinical evidence for poor albumin penetration in liver metastases has been observed using an albumin-binding positron emission tomography probe with binding affinity similar to dabrafenib (*58*). These observations and the findings presented suggest that the impact of albumin binding on effective free drug-target binding, especially in vivo, should be further explored.

Overall, this work presents KI lipophilicity and albumin binding as particularly problematic in treating poorly vascularized visceral tumors. Clinical imaging to quantify tumor vascularization and permeability is available (*50*), and clinical albumin imaging agents (*58*–*61*) may identify lesions with heterogeneous albumin extravasation (*60*, *62*) to guide treatment with highly protein-bound drugs (*63*). Such approaches, or even imaging with recently described KI radiotracers (*64*–*66*), may be integrated within a quantitative systems pharmacology framework to guide dosing, treatment selection, and possible combination therapy (*67*, *68*). In the case of BRAF-mutant cancers, such image-guided considerations may help a clinician weigh trade-offs between related KIs such as encorafenib and dabrafenib.

## MATERIALS AND METHODS

### Study design

The objective of this study was to understand how features of the tumor microenvironment including vascular permeability, functional perfusion, tumor size, and anatomical location influence PK/PD behaviors of KI in BRAF-V600–mutant cancers. Experiments were conducted with ≥3 independent replicates or as described in the figure captions; data collection and treatment group assignment were predetermined; no outliers were excluded. Previous studies and corresponding power analyses informed group sizes of this report (*69*). Analyses across treatment groups were performed blinded to treatment identity where possible; image acquisition, algorithms, and postprocessing were applied across whole images and groups with unbiased parameters. Complete materials and methods are included in the Supplementary Materials.

### Animal studies

All animal research was performed in accordance with guidelines from the Institutional Subcommittee on Research Animal Care and with approval of the Institutional Animal Care and Use Committee at Massachusetts General Hospital and Mayo Clinic. PDX experiments were conducted in accordance with the Belmont Report and U.S. Common Rule with approval from the Mayo Clinic Institutional Review Board and written consent from participating patients. Female mice aged 4 to 10 weeks were used for all studies, with B6129SF1/J (The Jackson Laboratory; for anaplastic thyroid cancer model), male C57Bl/6 (The Jackson Laboratory; YUMM1.7), nu/nu (Massachusetts General Hospital Cox7; A375, A375R, HT29, YUMMER1.7, PtD, and ES2), nu/nu (The Jackson Laboratory; A375 formalin-fixed paraffin-embedded experiments), and nu/nu (Envigo; PDX M12). Body condition score of 2 or less, weight loss exceeding 20%, and overt signs of pain or distress were among the criteria for euthanasia and humane survival.

### Retrospective clinical analysis

Retrospective clinical analysis was performed in accordance with the provisions of the Declaration of Helsinki and Good Clinical Practice guidelines. The Dana-Farber Cancer Institute/Harvard Cancer Center institutional review board deemed this study exempt. In summary, 81 patients and 96 total BRAFi/MEKi treatment courses were examined, who received treatment between 2010 and 2020 at Dana-Farber Cancer Institute and Massachusetts General Hospital Boston, Massachusetts (some patients were analyzed for both first and second courses of BRAFi/MEKi therapy). Inclusion/exclusion criteria and analysis details are outlined in the Supplementary Materials.

### Statistics and computational modeling

Image quantification was performed using Fiji/ImageJ (*70*) or CellProfiler v3.1.9 (in vitro ERK-KTR imaging) (*71*). Data analysis was performed using MATLAB R2017a (MathWorks, Natick, MA) and PRISM v8 (GraphPad, San Diego, CA). Log-linear analysis was performed as described (*72*). Statistical tests are indicated in figure captions and were two-tailed with α = 0.05 *P* value threshold. Multicompartmental modeling was performed in MATLAB R2017a (MathWorks, Natick, MA) using the method of lines. Analogous ordinary differential equations were solved as a homogeneous single compartment system to model cell culture. Apparent permeability (*P*_app_) was determined from time-lapse intravital microscopy data using previously published equations (*73*).

## Supplementary Materials

### This PDF file includes:

Supplementary Text Figs. S1 to S15 Tables S1 to S6 References

## Notes

View/request a protocol for this paper from *Bio-protocol*.
