# Supplementary material for: Overcoming differential tumor penetration of BRAF inhibitors using computationally guided combination therapy
Source: Sci Adv. 2022 Apr 29;8(17):eabl6339. doi: 10.1126/sciadv.abl6339 (PMC9054019; doi:10.1126/sciadv.abl6339)
Supplement: Supplementary file 1 — Supplementary Text Figs. S1 to S15 Tables S1 to S6 References [file sciadv.abl6339_sm.pdf]

Science Advances
AAAS

Supplementary Materials for

Overcoming differential tumor penetration of BRAF inhibitors using computationally guided combination therapy

Thomas S. C. Ng, Huiyu Hu, Stefan Kronister, Chanseo Lee, Ran Li, Luca Gerosa, Sylwia A. Stopka, Danielle M. Burgenske, Ishaan Khurana, Michael S. Regan, Sreeram Vallabhaneni, Niharika Putta, Ella Scott, Dylan Matvey, Anita Giobbie-Hurder, Rainer H. Kohler, Jann N. Sarkaria, Sareh Parangi, Peter K. Sorger, Nathalie Y. R. Agar, Heather A. Jacene, Ryan J. Sullivan, Elizabeth Buchbinder, Hannes Mikula, Ralph Weissleder, Miles A. Miller*

*Corresponding author. Email: miles.miller@mgh.harvard.edu

Published 29 April 2022, *Sci. Adv.* **8**, eabl6339 (2022)
DOI: 10.1126/sciadv.abl6339

This PDF file includes:

Supplementary Text
Figs. S1 to S15
Tables S1 to S6
References

Supplementary Text

Materials and Methods

Cells and reagents. SK-MEL-28, A375, and BRAFi-resistant A375R cell lines were from Dr. Hensin Tsao, and PtD were from Dr. David Pepin (both Massachusetts General Hospital, Boston, MA, USA). YUMMER1.7 cells were from M. Bosenberg (Yale). ES2, and HT29 cells were from American Type Culture Collection (ATCC), as were YUMM1.7 and A375 subsequently transfected with 4_pPB_ERKKTRmTq2_H2BVenus_mCherryGeminin as previously described (*41*). The TBP-3743 (*TPO*-Cre^{ER}; *Braf* $^{tm1Mmcm/+}$; *Trp53*$^{tm1Brn/tm1Brn}$) *Braf*$^{V600E/WT}$*p53*$^{-/-}$ murine cell line model of anaplastic thyroid cancer is described previously(*74*). Cell lines were grown with 10% FBS (Atlanta Biologicals), 100 IU mL^{-1}penicillin,100 μg mL^{-1}streptomycin (Invitrogen), with incubation at 37°C and 5% CO_2. All cell lines have been reported with V600E-mutant BRAF (*75-78*) and were routinely tested for mycoplasma (PCR Mycoplasma Detection Kit, Applied Biological Materials Inc., Canada and MycoAlert mycoplasma detection kit, Lonza). Mouse cell lines underwent PCR analysis of mouse pathogens and mycoplasma (IDEXX Laboratories, Westbrook, Maine). Chemicals and solvents were from Sigma Aldrich unless otherwise indicated. Stock drugs were in dimethyl sulfoxide (DMSO) throughout. Parent dabrafenib and trametinib were from LC Labs (Woburn, MA); encorafenib was obtained from MedChemExpress (Monmouth Junction, NJ).

ERK-KTR (pLentiCMV Puro DEST ERKKTR-Clover) was a gift from Markus Covert (Addgene plasmid # 59150) (*24*). Cell lines in some cases co-expressed nuclear markers including with the construct pLV-H2B-iRFP as described previously (*79*), and in the case of ES2 intravital imaging, JNK-KTR (pENTR-JNKKTRmRuby2), which was a gift from Markus Covert (Addgene plasmid #59148) (*24*). Cells were selected with puromycin and sorted through the Center for Regenerative Medicine and Technology / Department of Pathology Flow Cytometry Facility at Massachusetts General Hospital. In intravital A375 experiments, cells stably expressing 4_pPB_ERKKTRmTq2_H2BVenus_mCherryGeminin were used, generated by co-transfecting pCMV_hyPBase with lipofectamine 3000 (Invitrogen)(*80*) at a 5:2 ratio (w/w) and selecting with puromycin (1 μg mL^{-1}) followed by two rounds of cell sorting with a BD FACS Aria II.

Patient-derived (PDX) models for intracranial metastatic melanoma were conducted via the Mayo Clinic Brain Tumor PDX National Resource (*81*); primary cultures of M12 PDX cell lines grown in stem cell media were used as prior.

In vitro culture and imaging. Cytotoxicity measurements were performed in 96-well format by plating roughly 5000 cells per well overnight, treating with a dose-response of drug or DMSO vehicle for 72 hr, assessing cell count using a resazurin-based assay according to manufacturer guidelines (PrestoBlue, ThermoFisher Scientific), and read with a Tecan Spark fluorimeter (Switzerland). In vitro ERK-KTR imaging was performed by seeding roughly 20,000 cells per well in an optical-bottom 96-well plate (Ibidi, Germany) overnight, treating with drug or DMSO vehicle for 2 hr, adding 1 μg mL^{-1} Hoechst 33342, and imaging 10 min later with a modified Olympus BX63 inverted microscopy system with environmental chamber and robotic stage.

Biochemical dab-SiR characterization of dabrafenib and dab-SiR. Binding affinities to $BRAF^{V600E}$ were assayed using the LanthaScreen® Eu Kinase Binding Assay (ThermoFisher Scientific) following the manufacturer's protocol. All reagents and $BRAF^{V600E}$ recombinant human protein (sold as $BRAF^{V599E}$, which is equivalent to $BRAF^{V600E}$) were purchased from ThermoFisher Scientific/Life Technologies. Low volume 384-well plates (Corning, NY, USA) were used and read out using a Perkin Elmer EnVision Multilabel Reader (Waltham, MA, USA). Assays were performed in triplicates.

Confocal microscopy. Confocal microscopy was performed using a FluoView FV1000MPE confocal system (Olympus America) with XLFluor 2x (NA=0.14, Olympus) or XLUMPLFLN 20x (NA=1.0, Olympus) objectives. Sequential fluorescence channel excitation was performed with 405-, 473-, 559-, and 635-nm diode lasers, DM405/473/559/635-nm dichroic beam splitters and SDM473/560/640-nm beam splitters for emitted light. Detection used BA 430- to 455-nm, BA 490- to 540-nm, BA 575- to 620-nm, and BA 655- to 755-nm emission filters (Olympus). As an exception, mTurquoise2 / Venus /mCherry were imaged in A375 cells using an Argon-ion laser at 458 nm and 515 nm and with a diode laser at 559 nm using a DM 404-458/515/559-561

dichroic beam splitter. Emission light used a SDM510 dichroic beam splitter and a BA480-495 filter, SDM560 and a BA535-565 filter, and a BA575-675 filter.

Ex vivo tumor microscopy. Subcutaneous inoculation used 10^6 cells in 50 μL PBS, and tumors across all models were imaged upon reaching a diameter of 2.4 ± 0.9 mm (mean ± s.d.), generally 2-3 weeks post-inoculation. Pulmonary metastasis models used 1.25 x 10^5 cells injected via tail-vein catheter in 100 μL PBS, imaged 3-5 weeks post-inoculation. The model of melanoma metastasis to the liver used intrasplenic injection, whereby 5 x10^5 cells in 20 μL PBS were injected through a surgical incision over the spleen, which was closed by suture. Analgesic buprenorphine was given by intraperitoneal injection 30 min prior to surgery and every 6-12 hr thereafter for 72 hr; imaging was performed 10-15 days later. The intracranial melanoma model used 1.75 x 10^5 cells injected via intra-cardiac injection in 100 μL PBS, imaged 2 weeks post-inoculation(*82*). Disseminated intraperitoneal disease used inoculation with intraperitoneal injection of 5 x 10^6 cells in 200 μL PBS, with imaging roughly 10-14 days (ES2), 3-6 weeks (PtD), or 4-7 weeks (HT-29) later. For all metastasis models, imaging was performed upon detection of tumor burden by change in animal behavior (ambulation, breathing, posture), weight, body condition score, ascites, or palpation of tumor mass. Across all measurable metastasis models, tumor diameters were 2.9 ± 1.2 mm (mean ± s.d.). Where indicated, 50 μg DyLight 649 or rhodamine labeled Lycopersicon Esculentum (Tomato) Lectin (Vector Labs, Burlingame, CA) was given by tail vein injection in 50 μL PBS, 15 min prior to dissection. Fluorescently-labelled albumin was prepared as previously described*(52)*. Briefly, human serum albumin (HSA, Sigma) was conjugated to AlexaFluor 647 (A647, λex/λem = 650/665 nm) via N-hydroxysuccinimide (NHS) ester chemistry. 6.7 mg of albumin was dissolved in 100 μl sodium bicarbonate buffer (0.1 M, pH 8.2) and mixed with 1 mg of AlexaFluor NHS ester dyes (Invitrogen). The mixture was continuously stirred for 5 hours at room temperature at 300 r.p.m. Unreacted AlexaFluor dye was separated from the reaction mixture via centrifugation using 30 kDa molecular-weight cut-off (MWCO) Amicon centrifugal filter units (Millipore). Where indicated, this was given by tail vein injection (15 mg/kg, diluted into 100 μL PBS) 4 hrs prior to dissection.

Ex vivo tumor images were acquired immediately after excision from mice anesthetized under inhaled isoflurane on a heated mat, following terminal dissection surgery. 4 hr prior, mice were injected through tail-vein with 30 mg kg^{-1} dabrafenib and 10 mg kg^{-1} dab-SiR (where indicated) sonicated and vortexed in 120 μL solution of 5% DMAC:Solutol HS15, 10% DMSO, and 85% PBS. 10 mg kg^{-1} encorafenib was sonicated and vortexed in 120 μL solution of 5% DMAC:Solutol HS15, 10% DMSO, and 85% PBS and also injected intravenously. Quantification was performed in Fiji (ImageJ) (*70*) using line profiles in intensity extending radially into tumor center from the edge, or using hand-drawn regions of ~0.25 mm^2 at the tumor center compared to regions of similar size in adjacent non-tumor tissue. KTR readouts were interpreted from images acquired with the same acquisition settings, background-corrected before calculating C/N ratios for single-tumor cells.

In vivo microscopy. 2 x 10^6 ES2 reporter cells were injected under the fascia in 50 μL PBS within surgically implanted titanium dorsal skin-fold window chambers (APJ Trading). Analgesic buprenorphine was used prior to, and for 3 days after, window chamber implantation performed under 2% isoflurane with 2 L min^{-1} O_2. Sterile coverslips sealed the chambers and prophylactic antibiotic was supplied in drinking water. Upon tumor formation roughly 2 weeks later, mice were imaged following isoflurane anesthetization, tail-vein catheter placement, and immobilization on a 37°C microscope stage. Short-term BRAFi imaging (0-4 hr) in the ES2 model or in ear vasculature used 30 mg kg^{-1} dab-SiR supplied via catheter in sonicated and vortexed 100 μL solution of 10% DMAC:Solutol HS15, 10% DMSO, and 80% PBS. Ear imaging was performed on naïve female nu/nu mice by taping the ear to a heated stage and using otherwise identical intravital setups. KTR readouts were interpreted from images acquired with the same acquisition settings, background-corrected before calculating C/N ratios for single-tumor cells.

Transwell drug transport. A 2.5 mg/mL Collagen I (Collagen I, Rat Tail, Corning, NY) solution was prepared by diluting the stock solution into PBS and titrated with 1M NaOH to achieve pH 8.5. 100 μL of this solution was placed in the upper compartment of a 24-well translucent cell culture insert (0.45 μm pore size, high density, BD Falcon). The collagen gel was allowed to set

in an incubator at 37°C for at least 30 mins, and then both the upper and lower compartment of the transwell setup were filled with PBS (with or without 5% HSA) to prevent drying. A set of inserts without collagen was also prepared as a control. 100 μM solutions of dabrafenib and encorafenib were loaded in the upper compartment and stored in an incubator at 37°C over 48 hours. Samples from the upper and lower compartment of each insert setup was obtained at 5 hours post drug loading. The drug concentration between the upper and lower compartments were compared using high-performance liquid chromatography mass spectrometry (HPLC/MS) on a Waters instrument equipped with a Waters 2424 ELS Detector, Waters 2998 UV-Vis Diode array Detector, Waters 2475 Multi-wavelength Fluorescence Detector, and a Waters 3100 Mass Detector. Separations employed Waters XTerra RP C18 5 μm, with a water/acetonitrile solvent gradient (0.1% formic acid added). Drug concentration ratios between the upper and lower compartments for each condition were calculated from integrated $[M+H]^+$ and subsequently normalized to the average ratio detected in the condition lacking both HSA and Col I for each drug. Each condition was repeated at least 5 times over different days.

Transwell albumin transport. Mouse tumor-associated fibroblasts (colon cancer origin CT26, Cell Biologics Inc.) were grown in commercially sourced Fibroblast Medium (Cell Biologics Inc.). Cells were plated within the upper compartment of a 24-well translucent cell culture insert (0.45 μm pore size, high density, BD Falcon) at a concentration of 70 000 or 35 000 cells in 0.2 mL DMEM media containing 10% FBS, 100 IU mL^{-1}penicillin and 100 μg mL^{-1}streptomycin. After 2 days of incubation, fibroblasts were activated by replacing with fresh media containing 5 ng/mL(*83*) recombinant mouse TGF-β protein (Abclonal Technology) and incubated for an additional 4 days. At that time, media in both the upper (0.2 mL) and lower (0.8 mL) compartments were replaced, with the upper compartment now containing 5 µM AlexaFluor 647-HSA, prepared as described above. A set of inserts without fibroblasts served as a comparison, while wells without inserts containing media only and media mixed with molar equivalent AlexaFluor 647-HSA served as controls. HSA transport across the transwell were compared to controls at 60-, 90- and 180-minutes post-HSA incubation by aspiration of 50 µL aliquot samples from the lower compartment at each timepoint, and their fluorescence measured with fluorimetry in a 384-well plate. Immediately after these assays, all inserts were washed with

1x PBS, followed by fixation with 4% PFA. The collagen content present in each insert was then measured using a Sirius Red/Fast Green Collagen Staining Kit (Chondrex Inc). Collagen content was quantified, after liquid extraction, by measuring the absorbance at 540 nm, and corrected for non-collagen content by absorbance measured at 605 nm(*84*). Each condition was repeated 4 times.

Mathematical modelling of BRAFi tumor interstitial transport. A multicompartmental model was used to understand BRAFi PK/PD, with the assumption that only non-protein bound drug within the tissue compartment can interact with BRAF. Schematic of the model is depicted in Fig. 4A. Equations that describe the model are shown in Table S2. Input parameter values for dab-SiR, parent dabrafenib, and encorafenib are shown in Tables S3-4. Partial differential equations were solved using method of lines technique (*85*), as implemented in Matlab. Modeling of visceral metastases as seen on imaging (Fig. 5A, 8) was depicted as a spherical avascular lesion using the same equations described in Table S2 but adjusted to account for spherical geometry. For simplicity, combination drug modeling (Fig. 8) assumed independence except for competitive on-target BRAF binding. For directly comparing dabrafenib and encorafenib, k_{on} rate of the former was adjusted 7.5x higher to match relative biochemical binding affinity between the two drugs reported in Delord et al., 2017 (*2*).

Indirect comparison of clinical efficacy. Each of the three FDA-approved BRAFi/MEKi combinations for V600-mutant melanoma was compared to vemurafenib single-agent therapy, allowing for a cross-trial, indirect efficacy comparison. In the COMBI-v trial, D/T was compared to vemurafenib plus placebo, and was associated with an ORR of 64% compared to 51% in patients treated with single-agent vemurafenib. In the co-BRIM study, vemurafenib / cobimetinib (V/C) was compared with vemurafenib plus placebo. The ORR was 68% for the combination versus 48% for single-agent vemurafenib. The COLUMBUS trial compared E/B to single-agent vemurafenib (as well as to single-agent encorafenib). The ORR of combination therapy was 64% compared to 41% with single-agent vemurafenib in centralized review of imaging. It is important to note that the patient cohorts were well balanced and similar across trials (Figure S2D), although patients who received E/B were less likely to have an elevated LDH (29%) than patients who received D/T (34%) or V/C (43%). The method of Bucher *et al.* was applied in

which the indirect pairwise comparisons of D/T, V/C, or E/B are adjusted according to the results of the direct comparisons of each with the common control arm, vemurafenib[39]. Indirect efficacy comparisons of PFS and OS are expressed as relative hazard ratios (HR); indirect comparisons of ORR are expressed as relative risk ratios. All are accompanied by 95% confidence intervals. Inference is based on chi-squared tests.

Crystal structure visualization. Crystal structure visualizations of inhibitor-target complexes were visualized using the standard web browser viewer of rcsb.org (accessed 12-2019) and author-assigned assemblies for 5CSW, "B-RAF in complex with Dabrafenib" (*86*).

Statistics. Image quantification was performed using Fiji / ImageJ (*70*) or CellProfiler v3.1.9 (in vitro ERK-KTR imaging) (*71*). Data analysis was performed using Matlab R2017a (Mathworks, Natick, MA) and PRISM v8 (Graphpad, San Diego, CA). Log-linear analysis was performed as described (*72*), accessed 08-2020. Of note, this method does not allow for intra-patient covariation in lesion response to be accounted for, and lesions in the different individual anatomical sites (skin, lymph nodes, abdomen, etc.) were considered independent. This simplification motivated additional examination of intra-patient correlation in Fig. S14 using Fisher's exact test (see below). Statistical tests are indicated in figure captions and were two-tailed with $\alpha = 0.05$ p-value threshold.

Broad Repurposing Library data (PRISM Repurposing Secondary Screen 19Q4) was analyzed by calculating an AUC measurement by averaging reported viability values across all doses. Since same doses were used across drugs, and doses were evenly spaced in log-space (4-fold dilution), the AUC measurement is an area under the log-linear curve, normalized to a value of 1 if all viability measurements equal 1. Analysis was also performed using normalized integral procedures provided by depmap.org (*21*), yielding similar results (Fig. S15).

Retrospective Clinical Analysis. The retrospective clinical analysis was performed in accordance with the provisions of the Declaration of Helsinki and Good Clinical Practice guidelines. The Dana-Farber Cancer Institute / Harvard Cancer Center institutional review board deemed this study exempt (Protocol # 19-831). In sum, 81 patients and 96 total BRAFi/MEKi treatment courses were examined who received treatment between 2010-2020 at Dana-Farber Cancer Institute and Massachusetts General Hospital Boston, Massachusetts (some patients were analyzed for both first- and second- courses of BRAFi/MEKi therapy). Patients were selected who previously received a course of BRAFi/MEKi therapy, or who were BRAFi-naïve, and who had measurable tumor burden as guided by RECIST1.1 guidelines (≥1 cm lesion on long axis or ≥1.5cm short axis nodal lesion). All but one patient had confirmed BRAF mutation by central reference laboratory biopsy analysis (68/81 V600E; 4/81 V600K). The cohort was 48% male; stage (per American Joint Committee on Cancer 7th edition), age, and reasons for stopping BRAFi/MEKi are reported in Fig. S2A. Patients were excluded if they received concurrent oncologic treatment (e.g. immunotherapy) during the analyzed BRAFi/MEKi treatment course, underwent BRAFi/MEKi treatment for only 1 month or less, or if cross-sectional imaging was not obtained/available for review during the treatment course.

Retrospective review was performed of clinical imaging reports obtained before and during treatment. Radiologic imaging and associated reports interpreted as part of standard clinical practice were used to identify anatomical tumor location and response on a tumor-by-tumor basis. Baseline scan was defined as imaging performed prior to BRAFi/MEKi treatment onset. Imaging obtained throughout the BRAFi/MEKi treatment course was also analyzed. Radiologic response assessments often use Response Evaluation Criteria In Solid Tumors (RECIST 1.1) or similar, which simplify intrapatient heterogeneity of multifocal disease by pooling and summarizing measurements across organs (*87*). Here, RECIST 1.1 definitions for measurable disease were used to identify lesions, and RECIST 1.1 definitions for response (including 30% decrease in diameter denoting the threshold for partial response in tumors) were also used, however here sums of diameters were not computed across all organ sites as indicated in RECIST. 2-[18F]fluoro-2-deoxy-D-glucose (FDG) PET/CT (using the CT for lesion measurement), computed tomography of the chest, abdomen and/or pelvis, and magnetic

resonance imaging of the brain were analyzed. Lesions were identified on cross-sectional imaging for the following organ sites: skin, lymph nodes, lung, along the pleura, visceral abdomen and muscle. The long axis diameter of up to 2 target lesions in each organ (or in the case of lymph nodes, the short axis) were summed in the baseline scan. The same lesions were compared in follow-up studies. In most cases follow-up reports made size comparison with previous studies (and corresponding re-measurement of lesion diameter), and these data were used when available. Lesions at each organ site were categorized as showing response if the summed diameter decreased by ≥30%. Lesions at each organ site were deemed to have progressed if the summed diameter increased ≥20%, or if new lesions within the organ site were identified.

We hypothesized that BRAFi-naïve patients receiving D/T would show tumor-site bias for response compared to patients receiving E/B that had already received prior D/T. Across both treatment regimens, 40-55% of lesions responded to therapy depending on their anatomical tumor site, with the exception of tumors in the bone and brain, of which 11% and 33% responded, respectively. Due to limited sample size, lung and pleural lesions were pooled, skin and nodal lesions were pooled, and abdominal (primarily splenic and liver) lesions were pooled with intramuscular lesions. Bone and brain lesions were excluded due to limited sample sizes and biological distinction.

Intra-patient correlation in lesion response across tissue sites was found between skin and lymph node lesions, such that if skin lesions responded, lymph node lesions in the same patient were more likely to also respond (odds ratio, 12.8, 1.2-160 95% CI, n = 22 patients). However, less correlation was found when comparing response in either skin or lymph node lesions versus response in either abdominal or muscle lesions (Fig. S14; odds ratio, 2.3, 0.6-11 95% CI, n = 30 patients). Despite the limited sample sizes and potential tumor genetic confounders, this subset analysis further suggests response to BRAFi/MEKi depends on the tissue context.

Mass Spectrometry Imaging. YUMMER1.7 liver tumors were generated as in other experiments using intrasplenic injection, and subcutaneous tumors were also inoculated, with treatment performed approximately 2 weeks later. Intracranial PDX-melanoma metastases were generated as previously described (*88*) with 100 000 cells in 3 µL injected intracranially per mouse with treatment performed 12 days post injection. Mice were treated by intravenous injection of 30 mg kg^{-1} dabrafenib or 10 mg kg^{-1} encorafenib in 100 µL [PBS + 7% vol/vol DMAC:solutol + 10% DMSO]. 4 hr later, tumors were excised without perfusion and flash frozen by immediate cryovial submersion into liquid nitrogen. For drug quantitation with MALDI MSI, tissue mimetics were prepared using mouse brain homogenate spiked with the following drug concentrations for calibration: encorafenib 1.11-105.9 µM and dabrafenib 1.16-110 µM. The tissue mimetics were prepared for MALDI MSI using the same sample preparation workflow as the tissue specimens. Tissue specimens and mimetics were sectioned at 10 µm thickness, thaw mounted onto indium-tin-oxide (ITO) slides and placed in a desiccator until dry. Serial sections were stained with hematoxylin and eosin (H&E) and imaged by bright field microscopy (Zeiss Observer Z.1, Oberkochen, Germany) using a 20x plan-apochromat lens. The matrix solution consisted of 2,5- dihydroxybenzoic acid (160 mg mL^{-1}) dissolved in 70:30 methanol: 0.01% TFA with 1 % DMSO and was sonicated for 20 minutes. A TM-sprayer (HTX imaging, Carrboro, NC) sprayed the matrix onto the tissues sections with a two-pass cycle at flow rate 0.18 mL min^{-1}, spray nozzle velocity 1200 mm min^{-1}, spray nozzle temperature 75°C, nitrogen gas pressure 10 psi, and track spacing 2 mm. The matrix coated slide underwent recrystallization using 5 % acetic acid at 85°C for 6 minutes.

Imaging was performed using a timsTOFfleX mass spectrometer (Bruker Daltonics, Billerica, MA), and data were collected in positive ion mode within the m/z range of 100-2000. Images were acquired at 50 µm spatial resolution with the laser operating at 10 kHz for 1,000 shots per pixel. Prior to the imaging data acquisition, each drug solution was infused by electrospray ionization (ESI) to optimize the instrument parameters, e.g., ion transfer funnels, quadrupole, collision cell, and focus pre-TOF, and these values were then translated to the MALDI data acquisition method. Both drug methods were calibrated using the ESI source with an Agilent tune mix solution (Agilent Technologies, Santa Clara, CA). Full scan mode was implemented for

the quantification of dabrafenib and encorafenib from the liver samples by monitoring the $[M+H]^+$ ions. For improved sensitivity, multiple reaction monitoring (MRM) was used for the intracranial PDX-melanoma metastases models. For dabrafenib, the precursor to product transition monitored was 520.108→307.109 with the collision gas set to 43 eV and for encorafenib the MRM transition 540.162→359.118 with the collision gas set to 50 eV was monitored. Spectral data and ion images were visualized using SCiLS Lab software (version 2020a premium, Bruker Daltonics, Billerica, MA), and the dataset was normalized to the total ion current (TIC). Annotation and quantitation of encorafenib (540.1622 ± 5.8 ppm) and dabrafenib (520.1078 ± 1.0 ppm) were based on the $[M+H]^+$ ions.

In-vitro quantification of ERK inhibition in a BRAF-mutant cell line treated with multiple RAF and MEK inhibitors in presence or absence of Human Serum Albumin (HSA). Before being seeded at a density of 9000 cells/well in 96-well plates (Corning Cat#3603), A375 cells were resuspended in the same cultivation media supplemented with 1% FBS. Cells were grown in 96-well plates for 24 hr and then treated for 2 hr with the indicated RAF inhibitors dispensed using a Hewlett-Packard D300 Digital Dispenser and with Human Serum Albumin (HSA) (Sigma A1653) at a final concentration of 50 g/L or corresponding mock media volume by manual dispensing.

Cells were fixed in 4% PFA for 30 min at room temperature (RT) and washed with PBS with 0.1% Tween-20 (Sigma) (PBS-T), permeabilized in methanol for 10 min at RT, rewashed with PBS-T, and blocked in Odyssey blocking buffer (OBB LI-COR Cat. No. 927401) for 1 hr at RT. Cells were incubated overnight at 4 °C with primary antibody p-ERKT202/Y204 rabbit mAb (Cell Signaling Technology, clone D13.14.4E, Cat# 4370) in OBB. Cells were then stained with secondary antibodies from Molecular Probes (Invitrogen) labeled with Alexa Fluor 647 (Cat# A31573) at 1:2000 dilution. Cells were washed with PBS-T and then PBS and were next incubated in 250 ng/mL Hoechst 33342 and 1:2000 HCS CellMask™ Blue Stain solution (Thermo Scientific) for 20 min. Cells were washed twice with PBS and imaged with a 10× objective using an ImageXpress Micro Confocal High-Content Imaging System (Molecular Devices). 9 sites were imaged in each well for 96-well plates.

Quantification of p-ERKT202/Y204 was performed using Columbus imaging software (PerkinElmer). Cells contours were identified by a threshold on the CellMask™ Blue Stain Cells. Single-cell pERK intensity was quantified as the mean signal with the cell segmentation boundary. Mean intensity per well was calculated from all detected single-cells in that well, and mean overall p-ERK intensities and standard deviation per conditions were calculated from three well replicates using custom MATLAB 2017a code. pERK intensities within each 96-well plate were normalized by dividing the signal from untreated control cells.

Companion in-vitro microscopy assays were similarly performed using A375 and ES2 cells expressing the ERK-KTR. In vitro ERK-KTR imaging was performed by seeding roughly 20,000 cells per well in an optical-bottom 96-well plate (Ibidi, Germany), and treated as above with described concentrations of dabrafenib, dab-SiR and encorafenib. Cells were subsequently fixed with 4% PFA, followed by nuclear staining with 1 μg mL^{-1} DAPI, and imaging with a modified Olympus BX63 inverted microscopy system. KTR readouts were interpreted from images acquired with the same acquisition settings, background-corrected before calculating C/N ratios for individual tumor cells.

Efficacy in mice allografts. 5-7 week old male C57Bl/6 mice were subcutaneously inoculated with 250,000 YUMM1.7 $Braf^{V600E/wt}$ $Cdkn2a^{-/-}$ $Pten^{-/-}$ in 100 μl PBS. 24 days later, once tumors reached an average diameter of 6.4 ± 2.4 mm (mean ± s.d.), mice were randomly assigned to one of five treatment groups. Dosing was performed by oral gavage with 5 mg/kg encorafenib and/or 15 mg/kg dabrafenib administered in 100 μl water with 1% DMSO and 0.5% methylcellulose. Weight and tumor size were monitored daily. Independent sets of caliper measurements were made by two researchers, which were averaged to calculate volume according to the formula $V = 4/3\,\pi\,(0.5\,d)^3$, as in prior studies (*89*). To better control for differences in initial tumor size, tumor growth was calculated as a fraction of initial size. Minimum tumor size was set to 15 mm^3 across all groups during the fold-change calculation to control for caliper quantitation/detection limits. Tumors >10 mm in diameter, body condition score 2 or less, and tumor ulceration were all humane endpoint criteria. The last available caliper measurement was used in statistical calculation (Fig. 8C) if animals were sacrificed earlier due to humane endpoint consideration.

Chemical Synthesis and Characterization.

All reagents, unless otherwise noted, were purchased from commercial sources without further purification. Chloroform, methanol and THF were dried using PURESOLV- columns (Inert Corporation, USA). Dry DMF and DMSO were obtained from Sigma Aldrich. Solvents used for flash column chromatography were purchased from Donau Chemie AG (Austria). Reactions were carried out under an atmosphere of argon in air-dried glassware and magnetic stirring. Air- and/or moisture-sensitive liquids were transferred via syringe. Column chromatography was performed using a BUCHI Sepacore Flash System (2 x BUCHI Pump Module C-605, BUCHI Pump Manager C-615, BUCHI UV Photometer C-635, and BUCHI Fraction Collector C-660) or a Reveleris® X2 Flash Chromatography/Prep Purification System (BUCHI). Silica gel 60 (40-63 µm) was obtained from Merck and C18 flash cartridges for reversed phase chromatography were purchased from BUCHI (FlashPure Select C18, 30 µm) or Biotage (SNAP C18 Ultra, 25 µm). NMR spectra were recorded on a Bruker Avance IIIHD 600 MHz spectrometer equipped with a Prodigy BBO cryo probe, or on a Bruker Avance UltraShield 400 MHz spectrometer. Chemical shifts are reported in parts per million (δ) and calibrated using residual undeuterated solvent. Data are represented as follows: Chemical shift, multiplicity (s = singlet, d = doublet, t = triplet, q = quartet, m = multiplet, b = broad), coupling constant (J, Hz) and integration. HPLC analysis for reaction monitoring was performed either on a 1200 series system (Agilent Technologies, USA) using a Kinetex® (5 µm C18 100 Å, 50 x 4.6 mm, Phenomenex, USA) column, equipped with a DAD (Agilent Technologies) and a Bruker HCT Esquire Ion Trap MS, or on a Waters instrument (Waters 2424 ELS Detector, Waters 2998 UV-Vis Diode array Detector, Waters 2475 Multi-wavelength Fluorescence Detector, and a Waters 3100 Mass Detector). A Waters XTerra MS C18 column was used for separations. Routine analysis was conducted with 0.1% formic acid added to both solvents.

Dabrafenib-SiR (Dab-SiR)

Fig. S1. Scheme of Dab-SiR synthesis route.

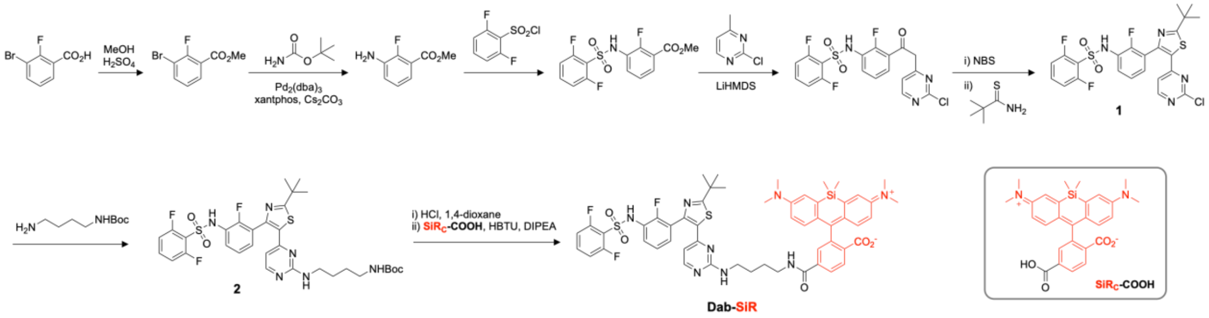

Dab-C_4-NHBoc (2)

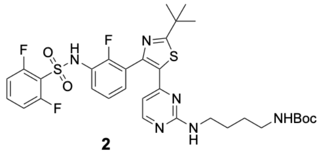

2

Compound 1 (prepared according to Rheault et al.*(90)*) (539 mg, 1 mmol) and N-Boc-1,4-diaminobutane (1.51 g, 8 mmol) were dissolved in 2-propanol (15 mL) and the solution was heated at 60 °C for 24 h. The solvent was evaporated and the residue was purified by reversed phase column chromatography (C18-silica, acetonitrile in water, gradient elution, 5-95% acetonitrile, eluents containing 0.1% formic acid) to obtain Dab-C_4-NHBoc (2) as a yellowish solid (355 mg, 51%); ^{1}H NMR (400 MHz, DMSO-d_6) δ 10.87 (s, 1H), 8.03 (d, J = 5.2 Hz, 1H), 7.78–7.59 (m, 1H), 7.44 (t, J = 7.6, 1.9 Hz, 1H), 7.33 (t, J = 6.7 Hz, 1H), 7.30–7.17 (m, 4H), 6.78 (t, J = 5.6 Hz, 1H), 5.92 (s, 1H), 3.16 (s, 2H), 2.94 (q, J = 6.5 Hz, 2H), 1.59–1.45 (m, 4H), 1.41 (s, 9H), 1.37 (s, 9H); ^{13}C NMR (101 MHz, DMSO-d_6) δ 181.8, 170.8, 162.5, 160.5, 160.5, 157.9, 157.9, 156.1, 154.7, 152.2, 145.8, 136.2, 134.9, 125.3, 113.9, 113.9, 113.7, 113.7, 105.2, 77.8, 60.2, 30.8, 28.7, 27.6, 26.7, 21.2; ESI-MS $[M+H]^+$ m/z calcd. 691.2 for $C_{32}H_{38}F_3N_6O_4S_2^+$, found 691.2.

Dab-SiR

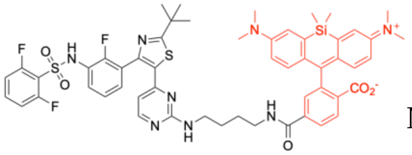

Dab-SiR

Compound 2 (34.5 mg, 0.05 mmol) was dissolved in anhydrous dichloromethane (1 mL) and a solution of HCl in 1,4-dioxane (4 M, 0.2 mL, 0.8 mmol) was added. The mixture was stirred at room temperature for 3 h and then concentrated. The obtained Boc-deprotected hydro-chloride was dissolved in dry DMF (0.1 mL) and DIPEA (17.4 µL, 0.1 mmol) was added (= solution 1). Separately, SiR_C-COOH[2] (28.4 mg, 0.06 mmol) was dissolved in dry DMF (0.5 mL) and DIPEA (17.4 µL, 0.1 mmol) and HBTU (22.8 mg, 0.06 mmol) were added, and the mixture was stirred at room temperature for 15 min (= solution 2). Solution 2 was then added to solution 1, and the mixture was stirred at room temperature for 18 h. The solution was directly loaded onto a C18 column (20 g). After reversed phase column chromatography (C18-silica, acetonitrile in water, gradient elution, 2-75% acetonitrile, eluents containing 0.1% formic acid) appropriate fractions (controlled by LCMS) were pooled and aqueous saturated NaHCO3 solution was added to adjust the pH to 7. Acetonitrile was evaporated (concentration to approx. half the volume) and EtOAc was added. The layers were separated and the aqueous layer was extracted twice with EtOAc. The combined organic layer was washed with brine, dried over

Na_2SO_4 and concentrated to afford Dab-SiR as a blue solid (35 mg, 67%); ^{1}H NMR (400 MHz, DMSO-d_6) δ 10.86 (s, 1H), 8.73 (t, J = 5.6 Hz, 1H), 8.17–7.96 (m, 3H), 7.71–7.60 (m, 2H), 7.42 (t, J = 7.4 Hz, 1H), 7.36–7.16 (m, 5H), 7.02 (t, J = 1.6 Hz, 2H), 6.69–6.60 (m, 4H), 3.31–3.09 (m, 4H), 2.91 (s, 12H), 1.52 (s, 4H), 1.37 (s, 9H), 0.64 (s, 3H), 0.53 (s, 3H); ^{13}C NMR (101 MHz, DMSO-d_6) δ 180.8, 169.8, 168.8, 164.2, 161.5, 159.5, 159.4, 156.9, 156.9, 154.4, 153.6, 151.2, 148.7, 144.8, 139.6, 135.3, 133.9, 129.9, 127.6, 127.1, 126.7, 124.8, 124.3, 122.2, 115.8, 113.2, 112.9, 112.7, 104.2, 90.7, 59.2, 37.1, 29.8, 26.0, -0.5, -1.8; ESI-MS $[M+H]^+$ m/z calcd. 1045.4 for $C_{54}H_{56}F_3N_8O_5S_2Si^+$, found 1045.4.

NMR spectra

Compound 2, ^{1}H NMR

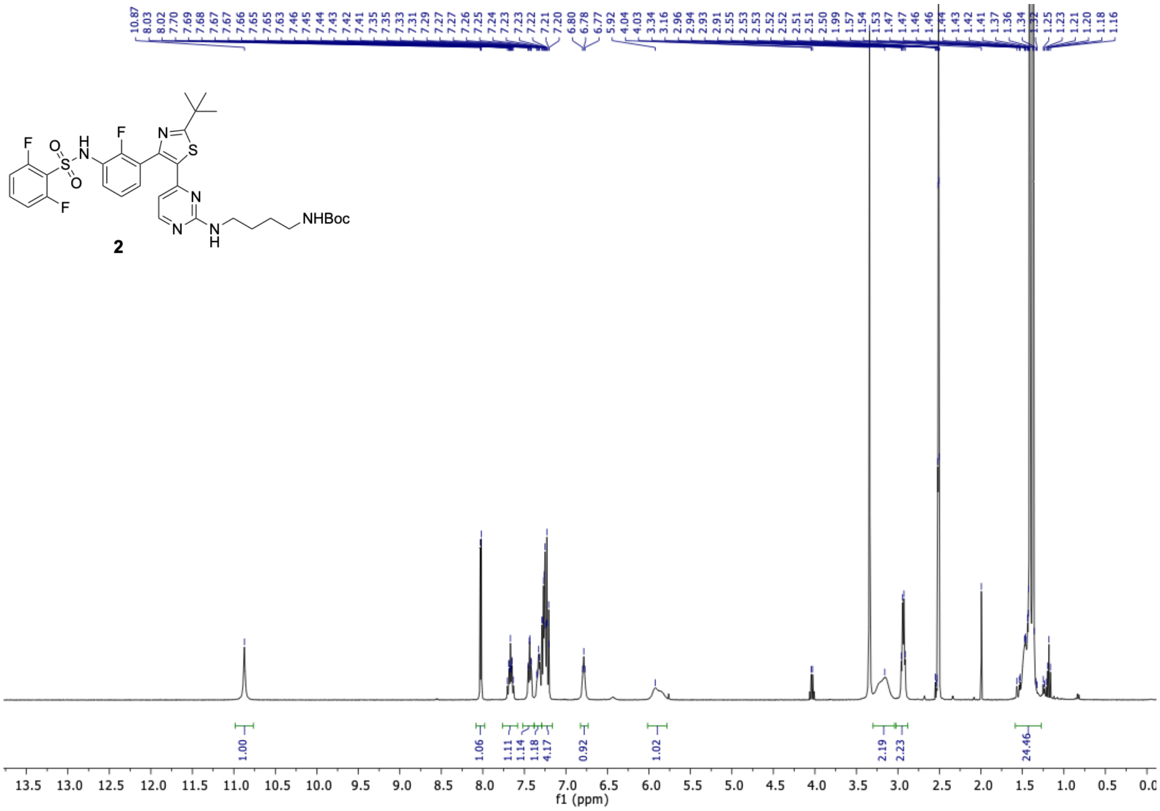

Compound 2, ^{13}C NMR

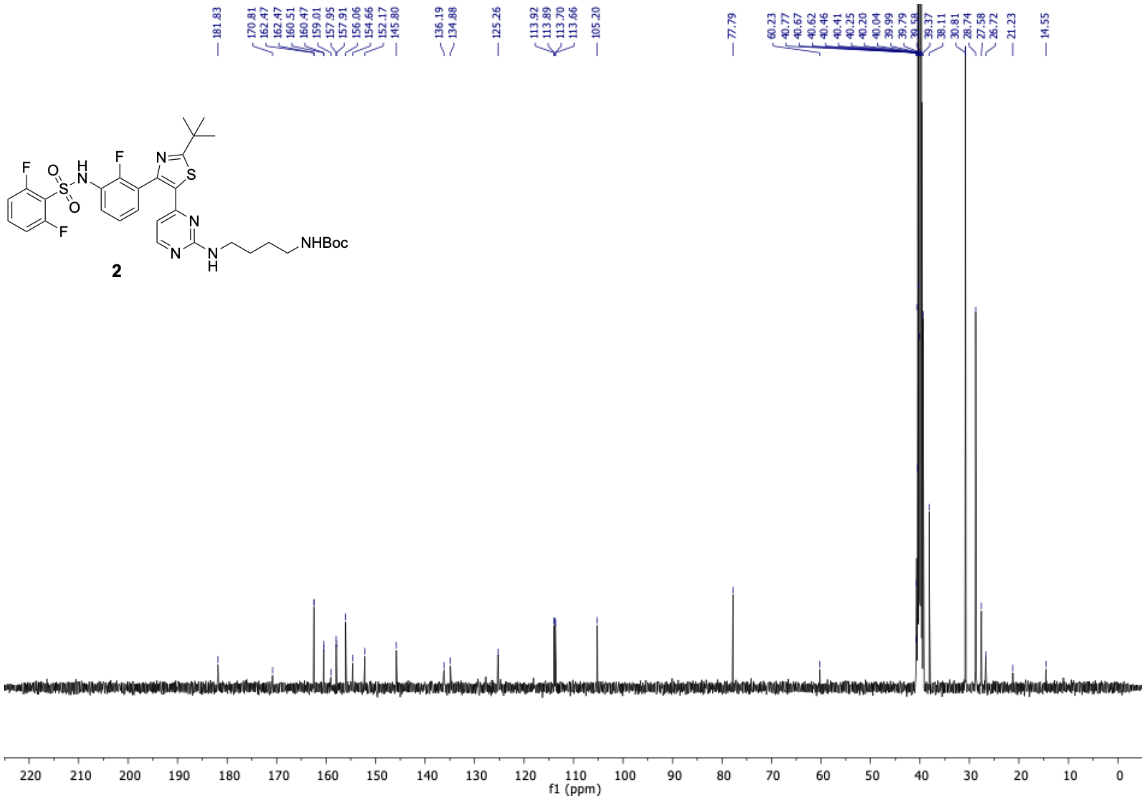

Dab-SiR, 1H NMR

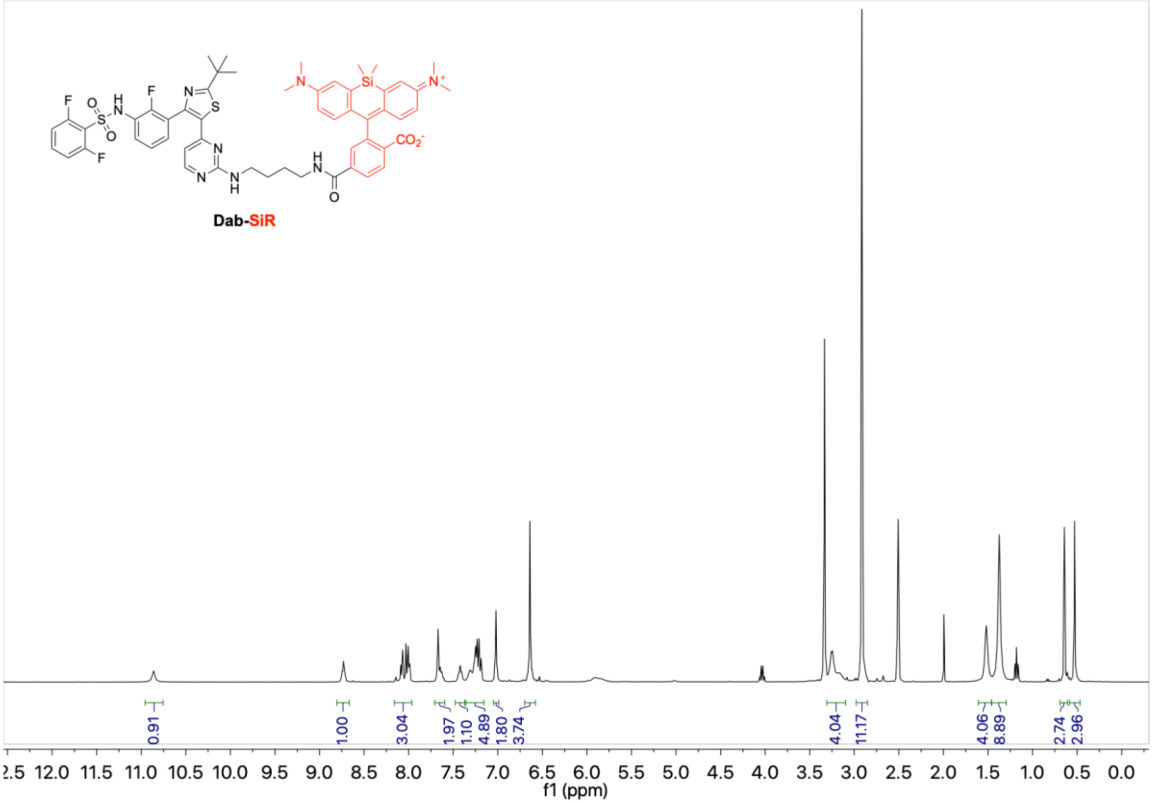

Dab-SiR, ^{13}C NMR

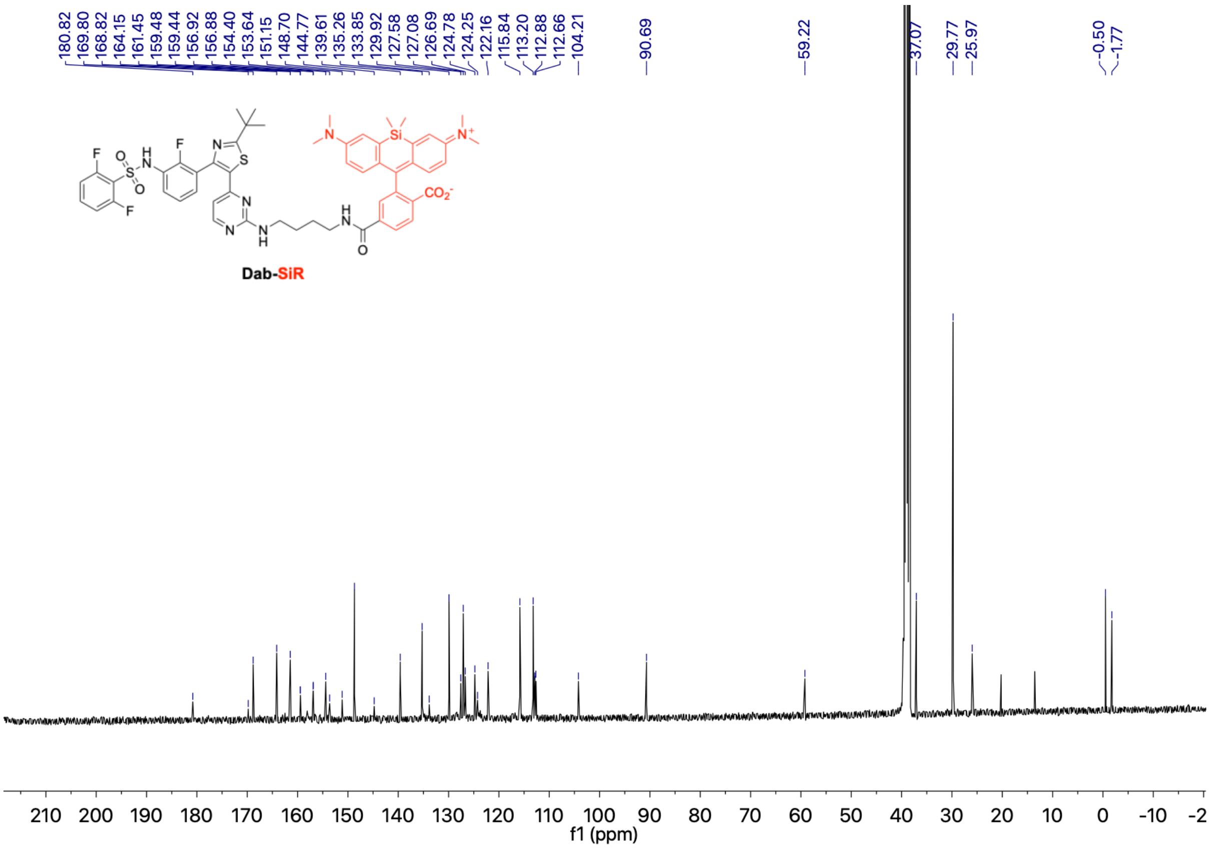

Fig. S2. **Retrospective clinical analysis summary. (A)** Summary of groups included (Fig. 1A-B). E/B, encorafenib / binimetinib. D/T, dabrafenib / trametinib. V/C, vemurafenib / cobimetinib. Reasons for stopping were counted when unambiguous. **(B)** Comparison of responses as in Fig. 1B, but only looking at groups A (left) and D (right) as defined in the table above. **(C)** Patients pre-treated with BRAFi/MEKi were categorized as exhibiting lesions with response at any organ site (as in Fig. 1B), and compared for the total duration of their prior course of BRAFi/MEKi. Data are means ± 95% CI (two-tailed Wilcoxon rank-sum test). **(D)** Indirect efficacy comparison of D/T, V/C, and E/B compiled from pivotal trial data. Progression free survival (PFS), overall survival (OS), and objective response rate (ORR) were calculated as means, 95% CI, and two-tailed significance tests. **(E)** Log-linear analysis corresponding to data in Fig. 1C.

A

group (size)	Pretreatment	Treatment	age (range)	stage	reason for stopping
A (61)	BRAFi/MEKi naïve	D/T	58 (21-90)	IIB (1/61)<br>III (16/61)<br>IV (44/61)	toxicity (22/61)<br>progression (12/61)<br>added/switched to immunotherapy (7/61)
B (5)	E/B (4/5)<br>V/C (1/5)	D/T	59 (41-72)	III (1/5)<br>IV (4/5)	progression (2/5)
C (13)	BRAFi/MEKi naïve	E/B	59 (25-76)	III (1/13)<br>IV (12/13)	toxicity (4/13)<br>progression/died (5/13)<br>added/switched to immunotherapy (1/13)
D (17)	D/T (17)	E/B	66 (30-83)	III (2/17)<br>IV (15/17)	progression/died (10/17)

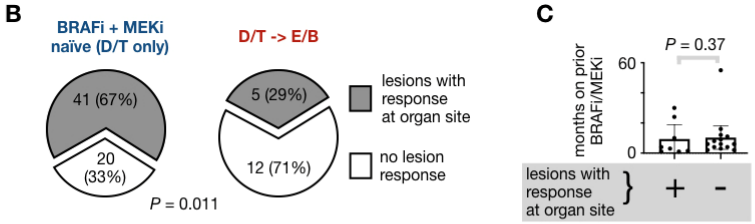

D

Trial characteristic	COMBI-v (D/T)	co-BRIM (V/C)	COLUMBUS (E/B)
median age (years)	55	55	57
gender (% male)	59	56	60
ECOG (%0)	71	67	71
Stage (AJCC 7th) (%)			
uStage III	4	5	5
M1a	16	16	14
M1b	17	17	18
M1c	63	62	64
LDH elevated (%)	34	43	29
BRAF status (%)			
V600E	90	70	89
V600K	10	13	11
unknown	0	17	0

Endpoint	D/T vs. V/C	D/T vs. E/B	V/C vs. E/B
PFS	1.00<br>(0.74–1.35)<br>$P = 0.99$	1.14<br>(0.81–1.61)<br>$P = 0.44$	1.14<br>(0.80–1.62)<br>$P = 0.46$
OS	0.94<br>(0.68—1.30)<br>$P = 0.72$	1.08<br>(0.77–1.51)<br>$P = 0.65$	1.15<br>(0.80–1.64)<br>$P = 0.45$
ORR	0.93<br>(0.76–1.12)<br>$P = 0.43$	0.82<br>(0.67–1.01)<br>$P = 0.07$	0.89<br>(0.71–1.11)<br>$P = 0.29$

E

log-linear analysis term	p-value
site *vs.* response	0.033
site *vs.* treatment	0.017
response *vs.* treatment	0.0008
3-way	0.009

Fig. S3. **Comparing in vitro kinase inhibitor effects. (A)**The ratio in concentration to inhibit 50% in a cytotoxicity assay (IC_{50}) was compared for sensitive and resistant cell lines (1-sample two-tailed wilcoxon test)(*2*). **(B)** The area under the curve (AUC) to in vitro cytotoxicity dose-response was compared across cell lines (yellow refers to models subsequently examined in this work)(*21*). Right, AUC was compared between drugs, such that a ratio 1 denotes equal cytotoxicity. **(C-D)** AUC ratios were calculated for vemurafenib, trametinib, and binimetinib. **(E-F)** AUC ratio comparisons across cancer cell types (left). No significant differences in AUC ratios were noted across different cancer cell types, regardless of BRAF mutation status. Skin and colorectal cancer comparisons plotted for reference (right). Data are individual cell lines, mean ± s.e. Two-tailed Wilcoxon rank-sum tests were performed.

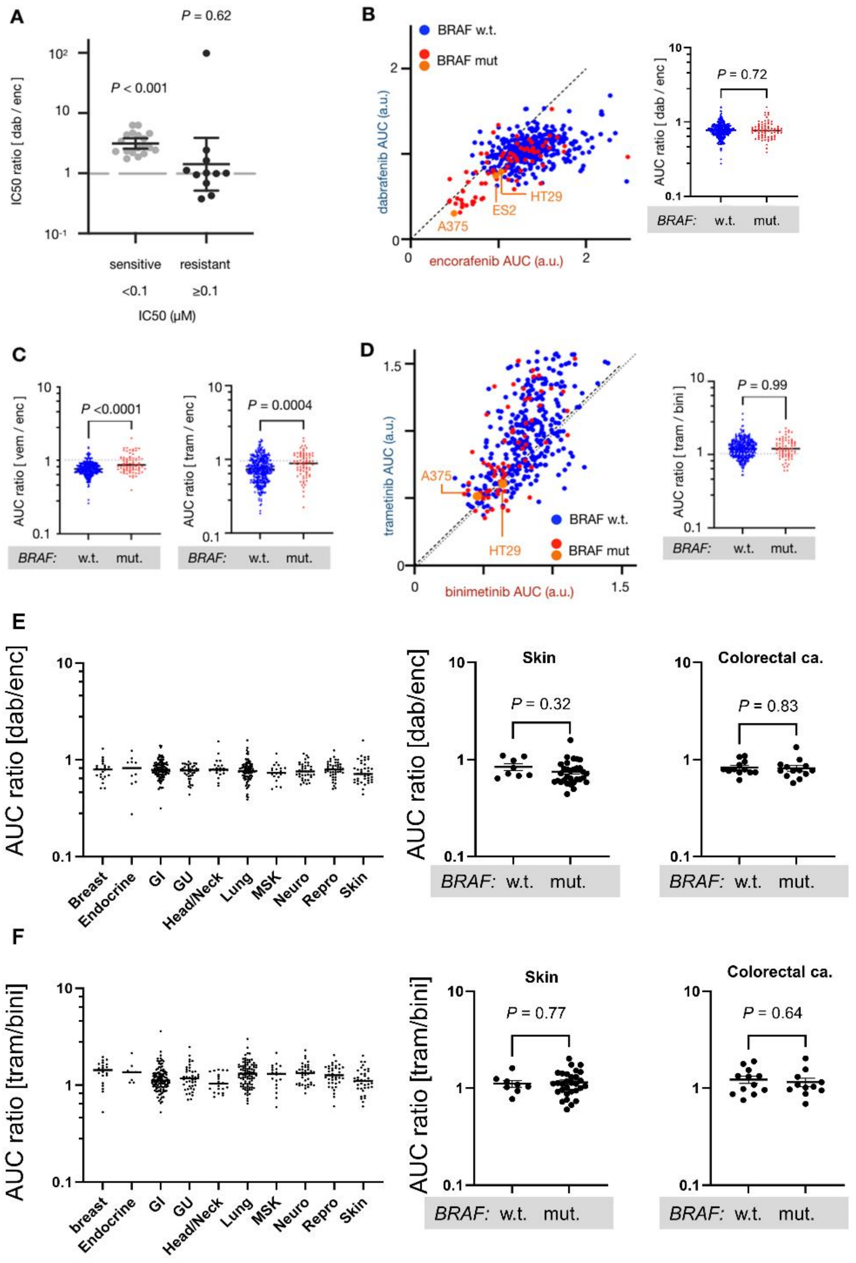

Fig. S4. Activity of dab-SiR. **(A)** The binding affinity (IC_{50}) of dab-SiR to purified recombinant V600E-mutant BRAF was determined and compared to binding of the parent dabrafenib (data are means ± s.e., n = 3). **(B)** Corresponding to data as in Fig. 2I, single-cell dab-SiR uptake was correlated with C/N activity readouts for ERK and JNK (Spearman rank correlation calculated across n = 30 cells per tumor, and then averaged across 3 tumors; two-tailed, one-sample wilcoxon test, means ± s.e.m). **(C)** Single-cells were classified by dab-SiR uptake and were compared over time for JNK activity (two-way ANOVA, n = 20 total cells per tumor, across n = 3 tumors).

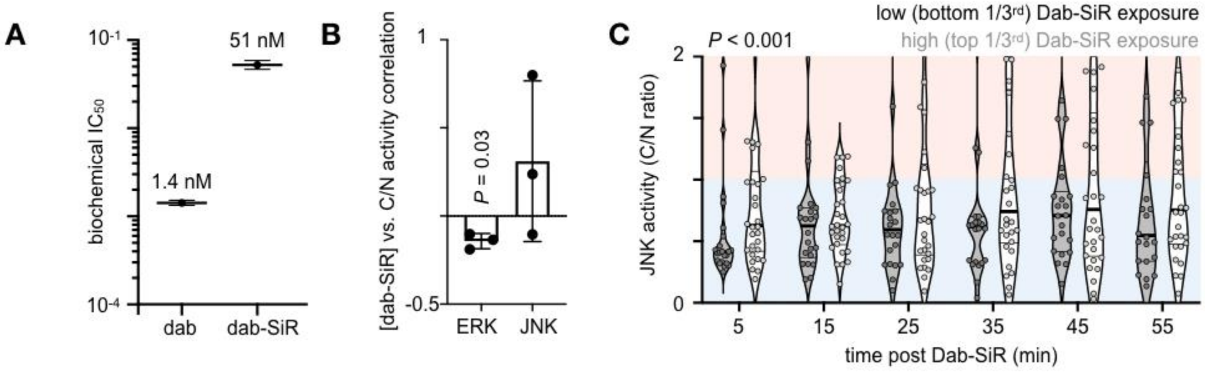

Fig. S5. Representative dab-SiR imaging and radial concentration profiles. Representative confocal microscopy and quantification of dab-SiR concentration as a function of radial distance from the tumor edge are shown. Thick line and shading denote means ± s.d. across n ≥ 2 tumors per model. In some instances, data are reshown from main figures (e.g. Fig. 3A for A375 model).

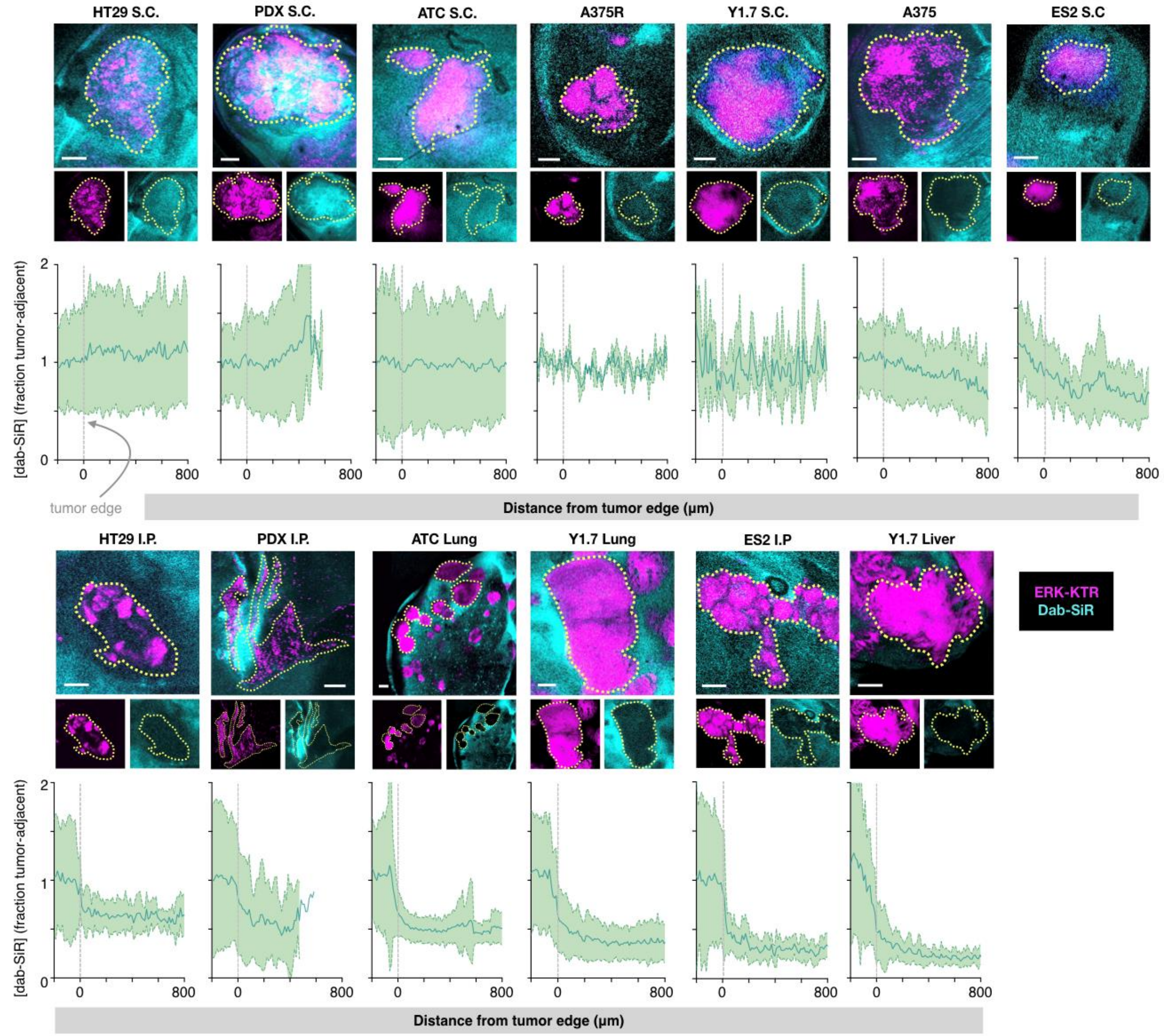

Fig. S6. Xenograft modeling of dab-SiR response. Corresponding to Fig. 3, diameters of tumors treated with dab-SiR are reported for matched subcutaneous and orthotopic models, showing no significant difference in size between the two at the time of imaging ($P = 0.50$, two-way ANOVA, $n = 49$ total tumors).

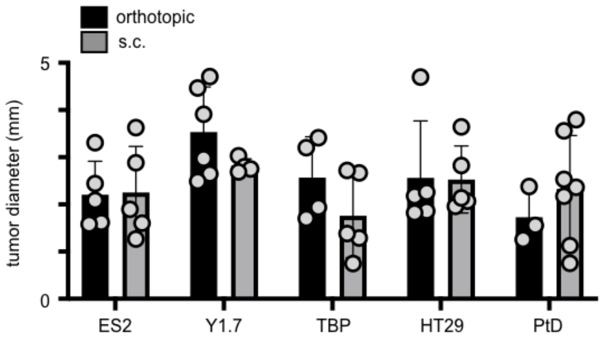

Fig. S7. Imaging of vasculature in subcutaneous and orthotopic tumor models. (A) Fluorescent lectin was injected prior to tumor excision to label tumor vasculature. Representative confocal microscopy and quantification of integrated lectin intensity as a function of radial distance from the tumor edge are shown. Thick line and shading denote means ± s.d. across n ≥ 2 tumors per model, with exception of YUMMER1.7 liver, measured once but over multiple line-profiles.

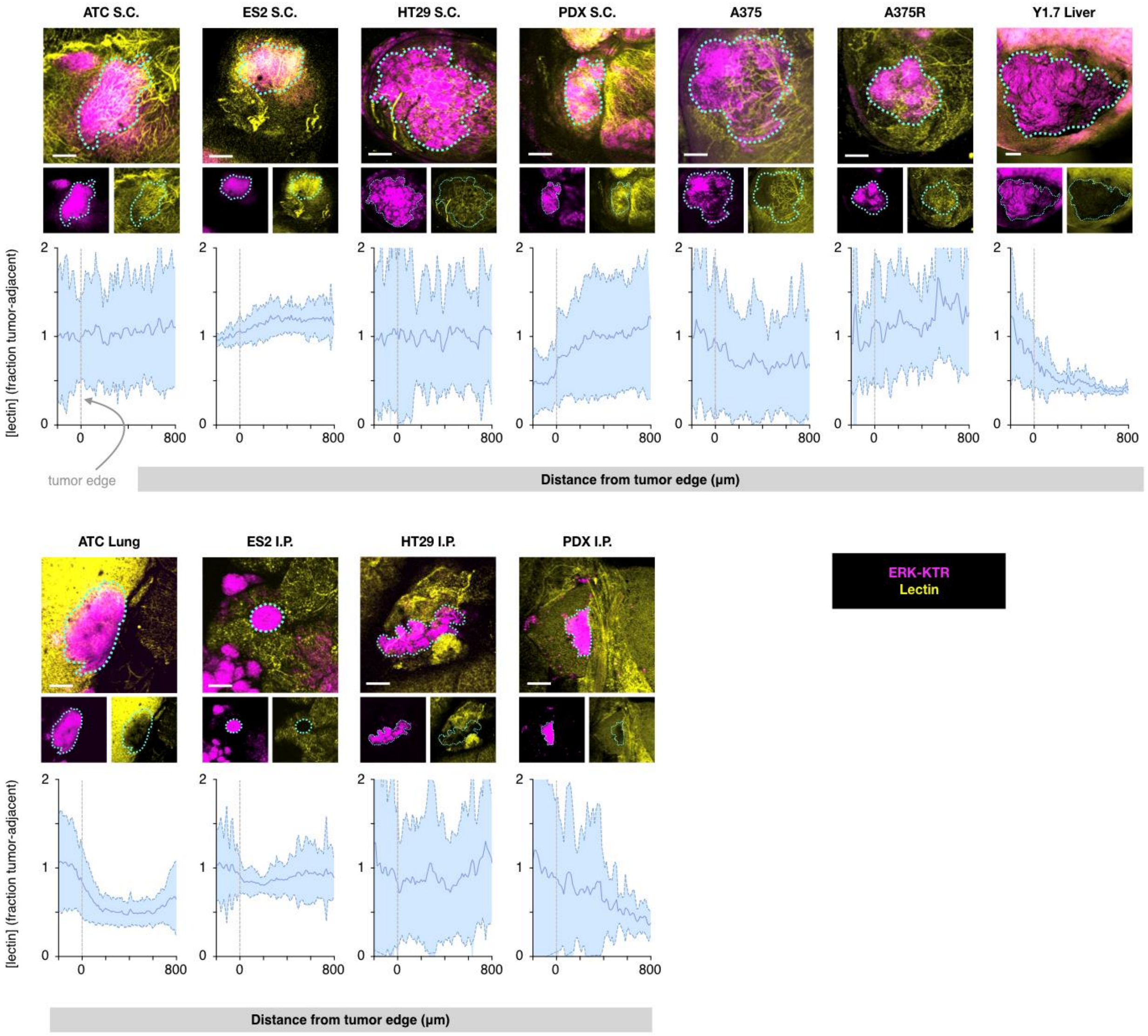

Fig. S8. In vitro dabrafenib cytotoxicity in YUMMER mouse melanoma cells. In a 96-well format, roughly 5,000 YUMMER1.7 cells per well were treated with a dose response of dabrafenib for 72 hr, and cell count was assessed by fluorescence microscopy immediately after DAPI counterstaining to label nuclei. The absolute IC_{50} and 95% confidence intervals are reported.

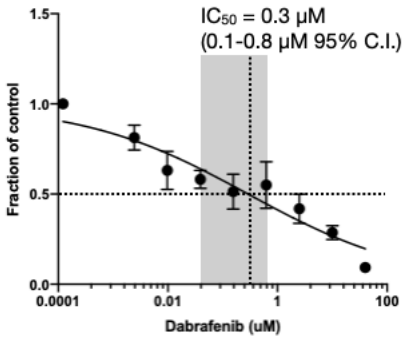

Fig. S9. Calibration curves for MALDI-MS. Concentration intensity calibration curves from tissue mimetic samples for dabrafenib (right) and encorafenib (left) across different runs – (**A**) for samples shown in Fig. 5, and (**B**) Fig. 7. Error bars denote standard deviation, each point derived from n = 3. (**C**) Representative calibrated mass spectrometry imaging (MALDI MSI) MRM of control intracranial tumor samples. Both drug measurements were below the threshold of detection for all control subjects (n = 5). Heme b measured as a marker for vasculature. Scale bar = 4 mm.

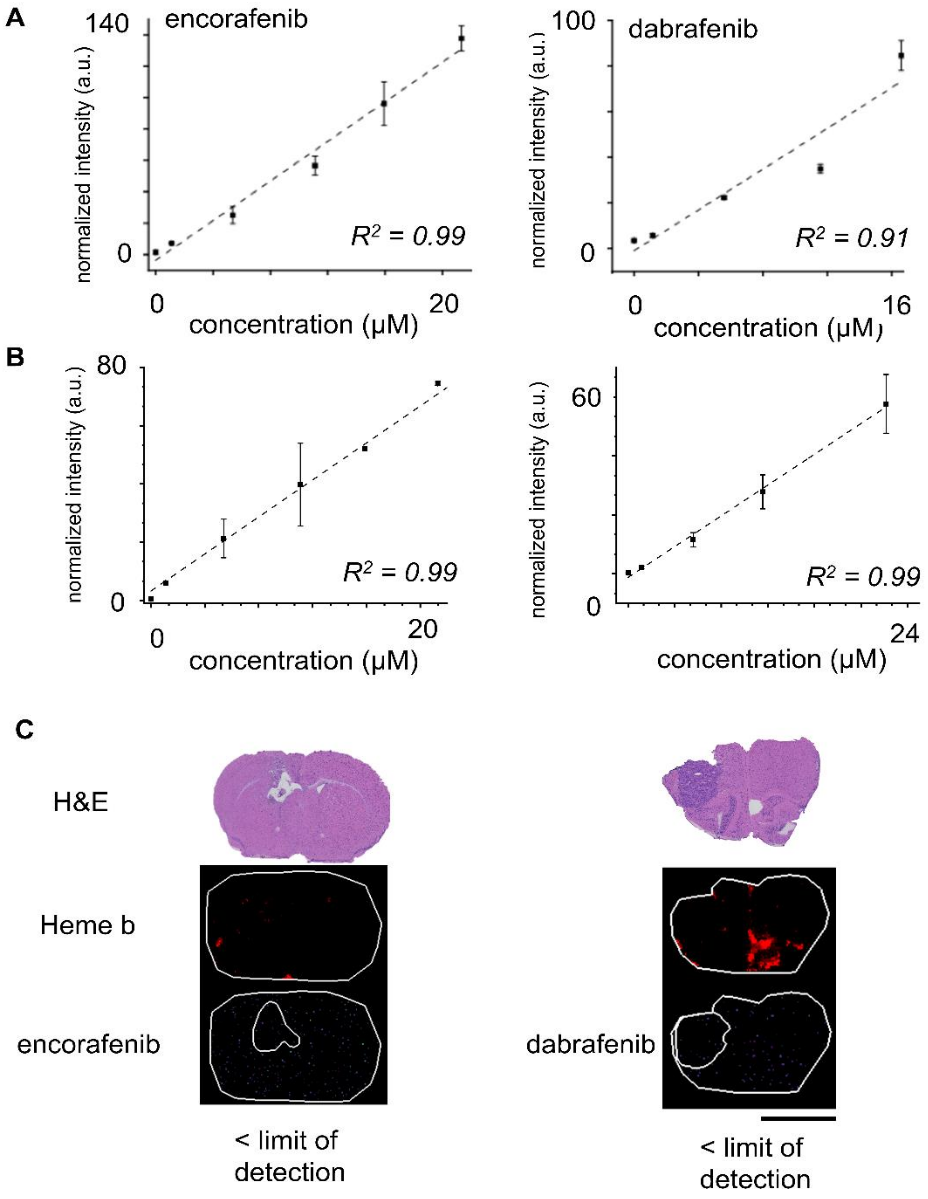

Fig. S10. Adjusted comparison of dabrafenib and encorafenib. (A) Cell line library cytotoxicity measurements were analyzed across a dose-response (data are 60 BRAF-mutant cell lines with median, Broad repurposing library). **(B)** Data from A were analyzed to compare effects at equimolar levels versus levels similar to as observed Fig. 5 (mean ± s.d. for 60 cell lines shown; two-tailed rank-sum test). Expected range of in vivo drug concentrations as measured from MALDI-MS, is highlighted with shaded boxes. **(C)** Simulated plasma drug concentration (top), tumor target occupancy (middle) and tumor concentration (bottom) for orally dosed dabrafenib and encorafenib based on human PK parameters and clinical dosing regimens. **(D)** Peak (+1hr) and trough drug concentration after 5 days of oral drug dosing, corresponding to C.

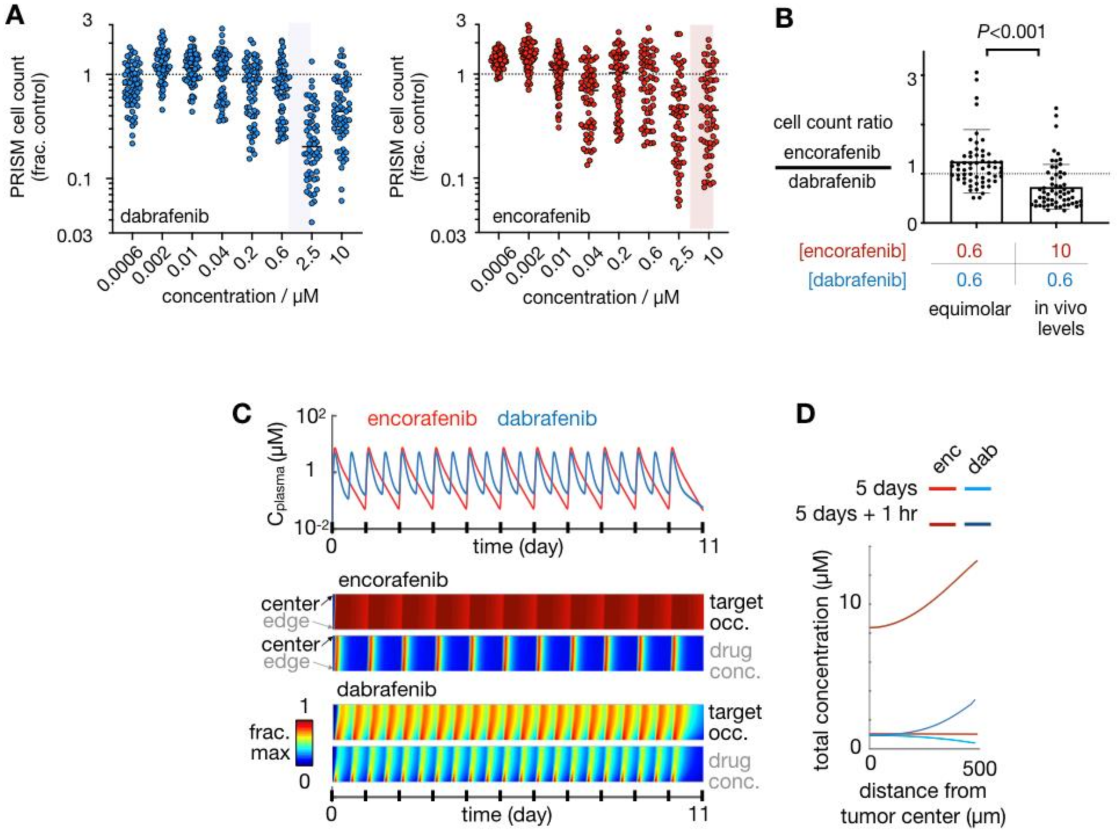

Fig. S11. Fibroblast-limited albumin transport. (A-B) Full dataset corresponding to Fig. 6D (which shows 180 min timepoint). A transwell cell-culture insert system in a similar configuration as described in Fig. 6A was used to assess albumin transport across a barrier of activated fibroblasts. AlexFluor 647-albumin transported into the lower well compartment was compared to controls, without an insert, for transwells prepared with and without activated mouse tumor-associated fibroblasts seeded at high (70 000 cells/well) and medium (35 000 cells/well) concentrations (mean ± s.e., analysis performed with a two-way ANOVA with repeated measures). **(B)** Collagen absorbance signal was quantified after Sirius red staining and liquid extraction (mean ± s.e., one-way ANOVA).

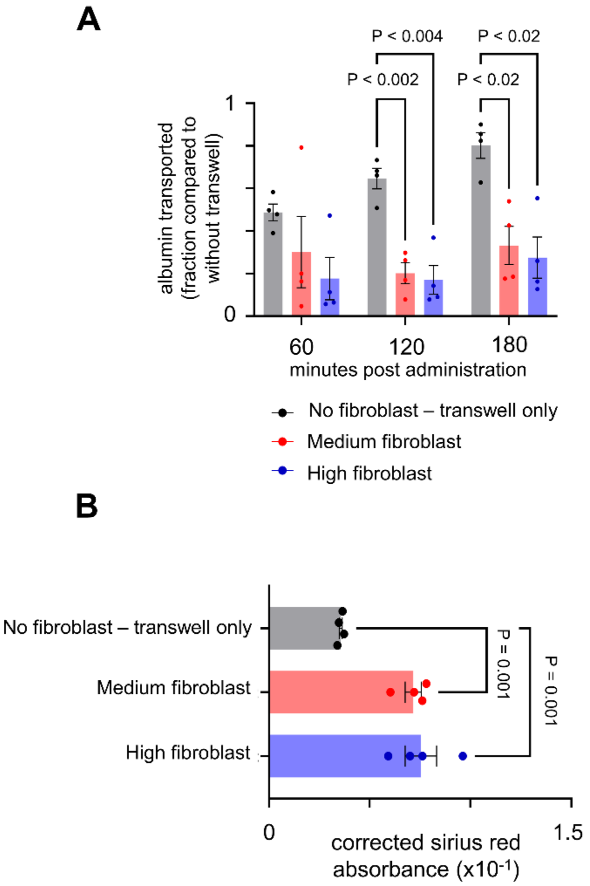

Fig. S12. In vitro assessment of dab-SiR activity as compared to native dabrafenib and encorafenib. (**A**) Quantification of dab-SiR cell uptake in the presence of albumin. (**B**) ERK-KTR activity of ES2 cells in the presence of dabrafenib, encorafenib and dab-SiR with and without albumin. Scale bar = 10 μm. Data from triplicate experiments. P-values from one-way ANOVA comparisons shown.

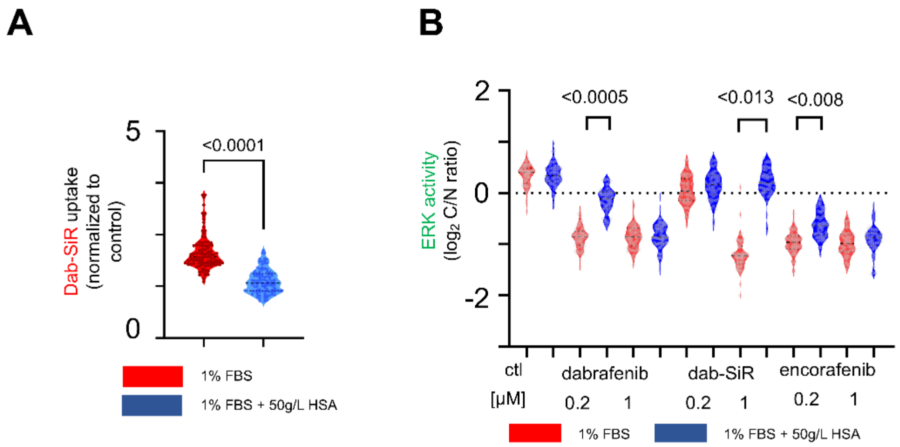

Fig. S13. KI tolerability and variability. (A) Computational assessment of BRAFi target occupancy and variance, as performed in Fig. 8A was performed for multiple single and combination treatment regimens. **(B)** Male C57Bl/6 mice were treated with BRAFi, and individual tumor growth measurements on day 9 were quantified for their initial response variability across the cohort (coefficient of variation, C.V. = std. dev. / mean). **(C)** Body weight was measured daily and no subjects lost >15% body weight. Data are means ± s.e.m. across n=19 total mice. **(D)** Indirect comparison of adverse events with at least 20% incidence in pivotal E/B and D/T trials among patients with advanced melanoma (see Fig. S1D). Data are means ± 95% C.I. **(E-G)** Nu/nu mice bearing hepatic YUMMER1.7 melanoma tumors were treated daily by oral gavage, body weight was monitored daily (F), and survival was monitored following guidelines for humane experimental endpoints (n=15 total mice across 4 groups; group colors match *F*). Images of excised livers, and subject-matched abdominal tumor mass in a dabrafenib-treated mouse, at time of sacrifice show relatively less tumor burden following 15 mg/kg encorafenib-treatment (E).

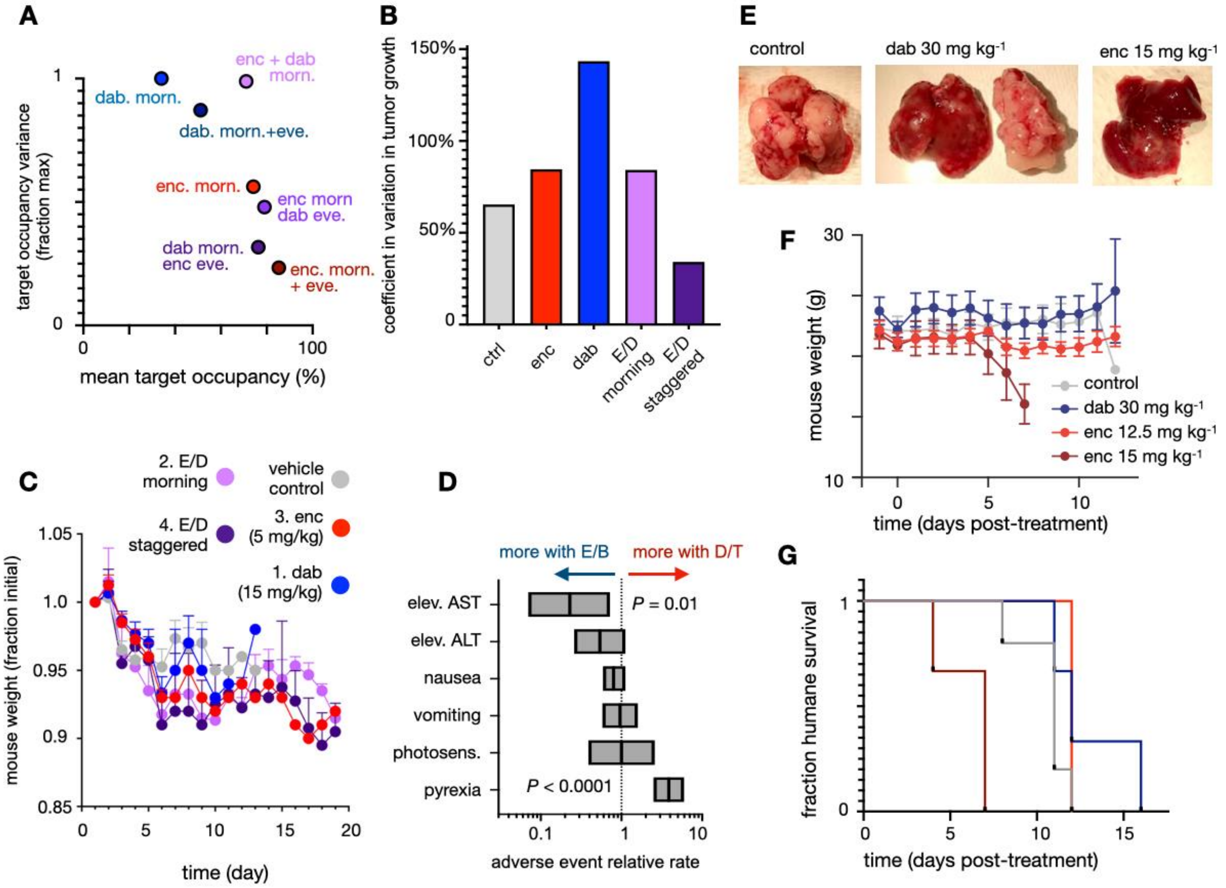

Fig. S14. **Analysis of intrapatient correlation in lesion response.** From patients receiving BRAFi/MEKi combination therapy (described in Fig. 1A-B), an analysis was performed to examine correlation in lesion responses across tissue sites. Responses (defined here as lesions shrinking >30% at the indicated anatomical site) were tabulated after first stratifying based on whether responses (as similarly defined) were noted at a separate indicated tumor site (the skin [in first comparison], or either skin and lymph node [second/third comparisons]). Odds ratios were then calculated (mean ± 95% CI) in comparing rates of lesion response depending on whether or not response was noted at the separate tumor site in the same patient and same course of BRAFi/MEKi. Data in table are patient counts, p-values in odds-ratios are from Fisher's exact test.

total n = 22

skin lesion response	no LN response	LN response
no	8	5
yes	1	8

total n = 21

skin or LN response	no lung/pleura response	lung/pleura response
no	7	4
yes	3	7

total n = 30

skin or LN response	no abd/musc response	abd/musc response
no	8	5
yes	7	10

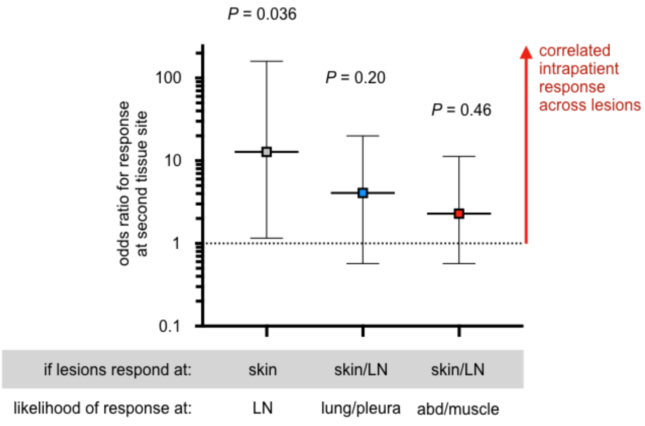

Fig. S15. Broad Repurposing Library alternative analysis. Analyses were performed as in Fig. S3, but using the depmap.org AUC calculation method of the normalized integral (*21*) rather than the normalized integral under the log-linear curve (see Methods). Despite differences in calculation, the AUC ratio trends were consistent (Fig. S3). Data are individual cell lines shown after standard depmap.org inclusion/filtering criteria (two-tailed rank-sum).

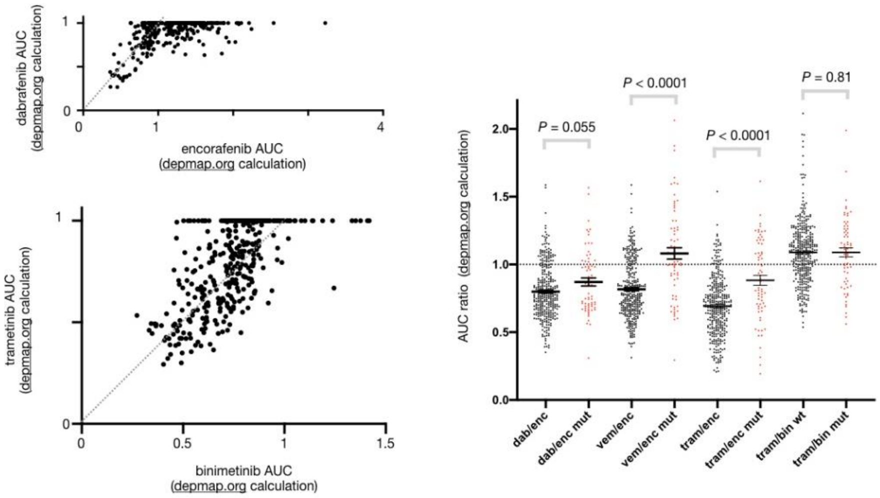

Table S1. Summary of drug PK properties. (A) Table comparing clinical pharmacokinetic parameters for BRAFi and MEKi, compiled from FDA package inserts and European Medical Authority (EMA) reports. **(B)** Summary of reported objective response rates (ORR) in BRAFi-pretreated patients with V600-mutant melanoma.

A

drug	target	combo	% protein bound	V_D (L)	terminal $t_{1/2}$ (hr)	CL (L hr^{-1})	Cmax (μM)	feces	urine	dose	BCS Class	XLogP
**dabrafenib (D)**	BRAF V600	D/T	99.7	70.3	8 hr*	17 (day 1) 34.4 (s.s.)	4.9	71%	23%	150mg BID	II	4.8
**vemurafenib (V)**	BRAF V600	V/C	99	106	57 hr	1.3	127	94%	1%	960mg BID	IV	5
**encorafenib (E)**	BRAF V600	E/B	86	164	3.5 hr	14 (d1) 32 (s.s.)	7.3	47%	47%	450mg QD	II	2.7
**trametinib (T)**	MEK1/2	D/T	97.4	214	3.9-4.8 d	4.9	3.3	80%	20%	2mg QD	II	3.4
**cobimetinib (C)**	MEK1/2	V/C	95	806	44 hr	13.8	0.5	76%	17.8%	60mg QD	I	3.9
**binimetinib (B)**	MEK1/2	E/B	97	92	3.5 hr	20	0.6	62%	31%	45mg BID	II	3.1

B

drug	response rate BRAFi-pretreated	prior BRAFi	response rate BRAFi-naïve	Reference
**E/B**	11/26 (42%)	D or V	28/42 (67%)	(91)
**E/B**	5/12 (42%)	not specified	10/14 (71%)	(5)
**E**	4/18 (22%)	97% V	9/15 (60%)	(2)
**D/T**	4/26 (26%)	46% V, 46% D		(92)
**D/T**	6/45 (15%)	100% D		(92)

Table S2. Pharmacokinetic model equations. Model corresponds to Fig. 4A.

Blood	
y_1 ub blood	$dy_1/dt = (k_{abs}\ y_2 + k_{abs2}\ y_4)\ V_P^{-1} - k_{el}\ y_1 - k_{on,ALB}\ y_1 + k_{off,ALB}\ y_3.$ if bolus, $y_1(t=0) = C_{max}$
y_2 ub gut	$dy_2/dt = D_1\ f_{DOSE1} - k_{abs1}\ y_2$ $f_{DOSE1} = bolus_{t=0}$, or $N(\mu, \sigma = 30\ min)$
y_3 bound blood	$dy_3/dt = k_{on,ALB}\ y_1 - k_{off,ALB}\ y_3 - k_{el}\ y_3$
y_4 ub gut, slow uptake	$dy_4/dt = D_2\ f_{DOSE2} - k_{abs2}\ y_4$ $f_{DOSE2} = N(\mu, \sigma = 30\ min)$
Transport	
y_5 tumor int., ub	$\partial y_5/\partial t = D_{eff,ub}\ (\partial^2 y_5/\partial r^2) + D_{eff,ub}\ r^{-1}\ (\partial y_5/\partial r)$
y_7 tumor int., alb bound	$\partial y_7/\partial t = D_{eff,ALB}\ (\partial^2 y_7/\partial r^2) + D_{eff,ALB}\ r^{-1}\ (\partial y_7/\partial r)$
Reactions	
y_5 tumor int., ub	$dy_5/dt = \textit{transport} - F_{nl}\ (k_{lu}y_5 - k_{lr}y_6) - k_{on,ALB}y_5 + k_{off,ALB}y_7 + k_{catab}y_8 + k_{off,ALB}y_8 - k_{on}y_5y_9 + k_{off}y_{10}$
y_6 tumor int., lipid bound	$dy_6/dt = k_{lu}y_5 - k_{lr}y_6$ *(no transport)*
y_7 tumor int., alb bound	$dy_7/dt = \textit{transport} + k_{on,ALB}y_5 - k_{off,ALB}y_7 - k_{mac}y_7$
y_8 tumor int., cellular ALB-bound	$dy_8/dt = k_{mac}y_7 - k_{catab}y_8 - k_{off,ALB}y_8$ *(no transport)*
y_9 tumor target binding sites	$dy_9/dt = -\ k_{on}y_5y_9 + k_{off}y_{10}$ *(no transport)*
y_{10} tumor bound target	$dy_{10}/dt = k_{on}y_5y_9 - k_{off}y_{10}$ *(no transport)*

Table S3. Description of model parameters. Corresponds to the model depicted in Fig. 4A with appropriate values pertinent for Dab-SiR modelling. These are presented alongside references from which the values were taken.

dab-SiR parameter	description	value	notes
R_{cap}	capillary radius	15 µm	Representative from intravital microscopy
V_p	cV_d (proportionate to volume of distribution)	n/a	Ratio with stomach PK; adjusted to fit observed dabrafenib PK (19)
$k_{el,1}$	fast-phase plasma elimination	0.004 min^{-1}	Representative from intravital microscopy, long circulating 3 hr half-life
$k_{el,2}$	slow-phase plasma elimination	n/a	Applied only for oral delivery
D_2	slow-phase contribution (fraction fast-phase)	n/a	Applied only for oral delivery
P_{ub}	permeability of tumor vasc., unbound drug	3×10^{-4} cm s^{-1}	From doxorubicin (range includes small molecule fluorophore conjugates), (93,94,107)
$D_{eff,\ ub}$	effective diffusion unbound drug	64×10^{-7} cm^2 s^{-1}	From fluorescein (95)
k_{lu}	partitioning into neutral lipid	10 min^{-1}	Highly variable but estimated from (96)
k_{lr}	partitioning into aqueous (lipid release)	k_{lu} / $D_{o:w}$	
log $D_{o:w}$	log_{10} water : octanol part. coeff., pH 7.4	4.65	estimated $logD_{dabrafenib}$ + 1
R_{krogh}	Krogh radius (R_{cap} + avg. intercap. dist.)	300 µm	poorly vascularized tumor
$D_{eff,\ ALB}$	effective diffusion albumin-bound drug	1×10^{-7} cm^2 s^{-1}	Modeled from (94, 95)
P_{ALB}	permeability of tumor vasc., albumin	2×10^{-7} cm s^{-1}	Modeled from (97-99)
$k_{on,ALB}$	albumin - drug association	10^6 M^{-1} s^{-1} · [ALB]	[ALB] = 500 µM
$f_{u,\ dab}$	fraction unbound dabrafenib (plasma)	0.004	(19), Dabrafenib FDA package insert
$K_{D,ALB}$	albumin - drug dissociation constant	2 µM	from f_u and [ALB]; estimated same as dabrafenib
$k_{off,ALB}$	albumin - drug dissociation	$k_{on,ALB}$ $[ALB]^{-1}$ $K_{D,ALB}$	
k_{mac}	cellular drug-albumin uptake	0.001 min^{-1}	(100-103)
F_{nl}	fraction neutral lipid	0.073	Fraction neutral lipid + 0.3 * fraction neutral phospholipid (104); estimated from melanoma biopsy (105)
k_{catab}	drug release from cellular ALB-drug complex	0.004 min^{-1}	From (52); little impact on model
k_{bind}	drug-target association	1.1 x 10^7 M^{-1} min^{-1}	Estimated from K_D and k_{off} (2, 26)
k_{off}	drug-target dissociation	0.3 min^{-1}	estimated as 50x dabrafenib k_{off} (see Fig. 4) (2)
DOSE	proportionate to dose	30 mg kg^{-1}	Little impact on model since scaled by Vp to fit C_{max} of dabrafenib (17)
T_{max}	time to maximum plasma concentration	n/a	oral delivery only
$\varepsilon_{unbound}$	void fraction free drug	0.4	(94)
ε_{bound}	void fraction bound drug (extracellular only)	0.2	(94)
[target]	concentration drug target	1 µM	(106)

Table S4. Input model parameter values. Data correspond to the different drugs and dose-administration pathways used in this study. These are presented alongside references from which the values were taken.

parameter	dab-SiR	dab (i.v.)	dab (p.o.)	*dab (p.o.) human*	enc (i.v.)	enc (p.o.)	*enc (p.o) human*	notes
$y_1(t=0)$	70 μM	70 μM	0	*0*	160 μM	0	*0*	dab-SiR and dab extrapoloated from (17); Enc i.v.: NDA 210496
V_p	n/a	n/a	400	*125*	200	12	*100*	Fit to plasma PK data
$k_{el,1}$	0.004 min^{-1}	0.03 min^{-1}	0.03 min^{-1}	*0.012* min^{-1}	0.012 min^{-1}	0.03 min^{-1}	*0.012* min^{-1}	Enc i.v.: NDA 210496
$k_{el,2}$	n/a	n/a	0.003 min^{-1}	*0.0014* min^{-1}	n/a	0.004 min^{-1}	*0.003* min^{-1}	Fit to plasma PK data
D_2	n/a	n/a	0.5	*0.2*	n/a	0.2	*0.9*	Fit to plasma PK data
log $D_{o:w}$	4.65	3.65	3.65	*3.65*	2.6	2.6	*2.6*	Dabrafenib was directly measured with mass spectrometry of octanol-water partitions, others were derived from online databases (https://go.drugbank.com/drugs/DB08911). Dab-SiR est. as 1 higher than Dab, based on mass spec
f_u	0.004	0.004	0.004	*0.004*	0.014	0.014	*0.14*	Enc i.v.: NDA 210496
k_{bind}	1.1 x 10^7 M^{-1} min^{-1}	1.1 x 10^7 M^{-1} min^{-1}	1.1 x 10^7 M^{-1} min^{-1}	*1.1 x* 10^7 M^{-1} min^{-1}	9.6 x 10^5 M^{-1} min^{-1}	9.6 x 10^5 M^{-1} min^{-1}	*9.6 x* 10^5 M^{-1} min^{-1}	variable (2, 108, 109)
k_{off}	0.3 min^{-1}	0.006 min^{-1}	0.006 min^{-1}	*0.006* min^{-1}	0.0004 min^{-1}	0.0004 min^{-1}	*0.0004* min^{-1}	Enc: (2)

Table S5. Analysis of simulated dabrafenib behavior in response to altered model parameters. Corresponds to Fig. 4F-H. Model rate constants were adjusted as shown, and compared to simulations of parent dabrafenib under conditions depicted in Fig. 4C.

	target fraction occupancy			target drug in tissue			
**model parameter**	**max proximal**	**max distal**	**mean proximal**	**mean distal**	**mean total drug (frac. parent)**	**distal/proximal peak total drug**	**notes**
**parent**	0.57	0.17	0.16	0.13	1.0	0.22	
**r_{krogh} = 100 um**	0.57	0.37	0.16	0.13	1.0	0.57	to model well-vascularized tissue
**no lipid partitioning**	0.57	0.18	0.16	0.14	0.9	0.3	
**Log $_{DO:W}$ 4.8**	0.57	0.15	0.16	0.11	2.6	0.16	high lipid partitioning
**$D_{eff} = 2 \times 10^{-8}$**	0.61	0.15	0.17	0.12	0.9	0.17	slow effective diffusion reported for vinblastine (110)
**no albumin binding**	0.99	0.99	0.92	0.98	73	0.16	
**10x f_u**	0.96	0.74	0.45	0.64	5.5	0.25	decreased albumin binding
**k_{mac} = 0**	0.57	0.17	0.16	0.13	1.0	0.22	no micropinocyto sis
**1/15th k_{off} & k_{on}**	0.23	0.15	0.18	0.12	0.9	0.32	K_d held constant (slower binding)
**15x k_{off} & k_{on}**	0.61	0.15	0.17	0.11	0.9	0.17	K_d held constant (faster binding)
**1/15th k_{off}**	0.89	0.73	0.75	0.54	3.3	0.57	K_d decreased by 15x (tighter binding)
**15x k_{on}**	0.97	0.75	0.52	0.56	3.4	0.52	K_d decreased by 15x (tighter binding)
**4x slower $t_{1/2}$**	0.80	0.47	0.40	0.38	3.1	0.38	

Table S6. Highly plasma protein bound oncology drugs. Drugs exhibiting ≥99% plasma protein binding and approved by the US FDA for indications in oncology are reported, along with their calculated water : octanol partition coefficient (cLogP) as a metric of lipophilicity (*54*).

cLogP	Drug	Notes
7.6	fulvestrant	intramuscular
6.9	bexarotene	hematological cancer(s)
6.8	venetoclax	hematological cancer(s)
6.4	tamoxifen	hormone targeted, pKa 8.7
6.3	toremifene	hormone targeted
5.5	dabrafenib	**
5.0	ponatinib	hematological cancer(s)
4.9	alectinib	pKa 7.05
4.7	cabozantinib	VEGFR targeted
4.6	lapatinib	pKa 7.2
4.6	vemurafenib	**
4.5	valrubicin	intracisternal administration
4.5	regorafenib	VEGFR targeted
4.1	axitinib	VEGFR targeted
4.0	abiraterone	hormone targeted
3.9	chlorambucil	hematological cancer(s)
3.9	vismodegib	basal cell skin cancer
3.5	pazopanib	VEGFR targeted
2.8	teniposide	hematological cancer(s)
2.7	ingenol	topical
2.6	ixazomib	hematological cancer(s)

REFERENCES AND NOTES

1. Z. Karoulia, E. Gavathiotis, P. Poulikakos, New perspectives for targeting RAF kinase in human cancer. *Nat. Rev. Cancer* **17**, 676–691 (2017).

2. J. Delord, C. Robert, M. Nyakas, G. McArthur, R. Kudchakar, A. Mahipal, Y. Yamada, R. Sullivan, A. Arance, R. Kefford, M. Carlino, M. Hidalgo, C. Gomez-Roca, D. Michel, A. Seroutou, V. Aslanis, G. Caponigro, D. Stuart, L. Moutouh-de Parseval, T. Demuth, R. Dummer, Phase I dose-escalation and -expansion study of the braf inhibitor encorafenib (LGX818) in metastatic BRAF-mutant melanoma. *Clin. Cancer Res.* **23**, 5339–5348 (2017).

3. V. Subbiah, I. Puzanov, J. Y. Blay, I. Chau, A. C. Lockhart, N. S. Raje, J. Wolf, J. Baselga, F. Meric-Bernstam, J. Roszik, E. L. Diamond, G. J. Riely, E. J. Sherman, T. Riehl, B. Pitcher, D. M. Hyman, Pan-cancer efficacy of vemurafenib in BRAF (V600)-mutant non-melanoma cancers. *Cancer Discov.* **10**, 657–663 (2020).

4. R. Corcoran, T. André, C. Atreya, J. Schellens, T. Yoshino, J. Bendell, A. Hollebecque, A. McRee, S. Siena, G. Middleton, K. Muro, M. Gordon, J. Tabernero, R. Yaeger, P. O'Dwyer, Y. Humblet, F. De Vos, A. Jung, J. Brase, S. Jaeger, S. Bettinger, B. Mookerjee, F. Rangwala, E. Van Cutsem, Combined BRAF, EGFR, and MEK inhibition in patients with $BRAF^{V600E}$-mutant colorectal cancer. *Cancer Discov.* **8**, 428–443 (2018).

5. R. Dummer, P. Ascierto, H. Gogas, A. Arance, M. Mandala, G. Liszkay, C. Garbe, D. Schadendorf, I. Krajsova, R. Gutzmer, V. Chiarion-Sileni, C. Dutriaux, J. de Groot, N. Yamazaki, C. Loquai, L. Moutouh-de Parseval, M. Pickard, V. Sandor, C. Robert, K. Flaherty, Encorafenib plus binimetinib versus vemurafenib or encorafenib in patients with BRAF-mutant melanoma (COLUMBUS): A multicentre, open-label, randomised phase 3 trial. *Lancet Oncol.* **19**, 603–615 (2018).

6. S. Kopetz, A. Grothey, R. Yaeger, E. Van Cutsem, J. Desai, T. Yoshino, H. Wasan, F. Ciardiello, F. Loupakis, Y. Hong, N. Steeghs, T. Guren, H. Arkenau, P. Garcia-Alfonso, P. Pfeiffer, S. Orlov, S. Lonardi, E. Elez, T. Kim, J. Schellens, C. Guo, A. Krishnan, J. Dekervel, V. Morris, A. Calvo Ferrandiz, L. Tarpgaard, M. Braun, A. Gollerkeri, C. Keir, K. Maharry, M. Pickard, J. Christy-Bittel,

L. Anderson, V. Sandor, J. Tabernero, Encorafenib, binimetinib, and cetuximab in BRAF V600E-mutated colorectal cancer. *N. Engl. J. Med.* **381**, 1632–1643 (2019).

7. H. Shi, W. Hugo, X. Kong, A. Hong, R. C. Koya, G. Moriceau, T. Chodon, R. Guo, D. B. Johnson, K. B. Dahlman, M. C. Kelley, R. F. Kefford, B. Chmielowski, J. A. Glaspy, J. A. Sosman, N. van Baren, G. V. Long, A. Ribas, R. S. Lo, Acquired resistance and clonal evolution in melanoma during BRAF inhibitor therapy. *Cancer Discov.* **4**, 80–93 (2014).

8. A. Hong, G. Moriceau, L. Sun, S. Lomeli, M. Piva, R. Damoiseaux, S. Holmen, N. Sharpless, W. Hugo, R. Lo, Exploiting drug addiction mechanisms to select against MAPKi-resistant melanoma. *Cancer Discov.* **8**, 74–93 (2018).

9. A. Minchinton, I. Tannock, Drug penetration in solid tumours. *Nat. Rev. Cancer* **6**, 583–592 (2006).

10. S. Wilhelm, A. Tavares, Q. Dai, S. Ohta, J. Audet, H. Dvorak, W. Chan, Analysis of nanoparticle delivery to tumours. *Nat. Rev. Mater.* **1**, 1–12 (2016).

11. Y. Sun, J. Alberta, C. Pilarz, D. Calligaris, E. Chadwick, S. Ramkissoon, L. Ramkissoon, V. Garcia, E. Mazzola, L. Goumnerova, M. Kane, Z. Yao, M. Kieran, K. Ligon, W. Hahn, L. Garraway, N. Rosen, N. Gray, N. Agar, S. Buhrlage, R. Segal, C. Stiles, A brain-penetrant RAF dimer antagonist for the noncanonical BRAF oncoprotein of pediatric low-grade astrocytomas. *Neuro Oncol.* **19**, 774–785 (2017).

12. S. Torok, M. Rezeli, O. Kelemen, A. Vegvari, K. Watanabe, Y. Sugihara, A. Tisza, T. Marton, I. Kovacs, J. Tovari, V. Laszlo, T. Helbich, B. Hegedus, T. Klikovits, M. Hoda, W. Klepetko, S. Paku, G. Marko-Varga, B. Dome, Limited tumor tissue drug penetration contributes to primary resistance against angiogenesis inhibitors. *Theranostics* **7**, 400–412 (2017).

13. E. Randall, K. Emdal, J. Laramy, M. Kim, A. Roos, D. Calligaris, M. Regan, S. Gupta, A. Mladek, B. Carlson, A. Johnson, F. Lu, X. Xie, B. Joughin, R. Reddy, S. Peng, W. Abdelmoula, P. Jackson, A. Kolluri, K. Kellersberger, J. Agar, D. Lauffenburger, K. Swanson, N. Tran, W. Elmquist, F. White, J. Sarkaria, N. Agar, Integrated mapping of pharmacokinetics and pharmacodynamics in a patient-derived xenograft model of glioblastoma. *Nat. Commun.* **9**, 4904 (2018).

14. J. Li, J. Wu, X. Bao, N. Honea, Y. Xie, S. Kim, A. Sparreboom, N. Sanai, Quantitative and mechanistic understanding of AZD1775 penetration across human blood-brain barrier in glioblastoma patients using an IVIVE-PBPK modeling approach. *Clin. Cancer Res.* **23**, 7454–7466 (2017).

15. J. Wang, C. Gan, R. W. Sparidans, E. Wagenaar, S. van Hoppe, J. H. Beijnen, A. H. Schinkel, P-glycoprotein (MDR1/ABCB1) and breast cancer resistance protein (BCRP/ABCG2) affect brain accumulation and intestinal disposition of encorafenib in mice. *Pharmacol. Res.* **129**, 414–423 (2018).

16. H. Ellens, M. Johnson, S. K. Lawrence, C. Watson, L. Chen, L. E. Richards-Peterson, Prediction of the transporter-mediated drug-drug interaction potential of dabrafenib and its major circulating metabolites. *Drug Metab. Dispos.* **45**, 646–656 (2017).

17. R. K. Mittapalli, S. Vaidhyanathan, R. Sane, W. F. Elmquist, Impact of P-glycoprotein (ABCB1) and breast cancer resistance protein (ABCG2) on the brain distribution of a novel BRAF inhibitor: Vemurafenib (PLX4032). *J. Pharmacol. Exp. Ther.* **342**, 33–40 (2012).

18. I. N. Okten, S. Ismail, B. M. Withycombe, Z. Eroglu, Preclinical discovery and clinical development of encorafenib for the treatment of melanoma. *Expert Opin. Drug Discov.* **15**, 1373–1380 (2020).

19. R. K. Mittapalli, S. Vaidhyanathan, A. Z. Dudek, W. F. Elmquist, Mechanisms limiting distribution of the threonine-protein kinase B-RaF^{V600E} inhibitor dabrafenib to the brain: Implications for the treatment of melanoma brain metastases. *J. Pharmacol. Exp. Ther.* **344**, 655–664 (2013).

20. F. Agnello, M. Ronot, D. C. Valla, R. Sinkus, B. E. Van Beers, V. Vilgrain, High-b-value diffusion-weighted MR imaging of benign hepatocellular lesions: Quantitative and qualitative analysis. *Radiology* **262**, 511–519 (2012).

21. S. M. Corsello, R. T. Nagari, R. D. Spangler, J. Rossen, M. Kocak, J. G. Bryan, R. Humeidi, D. Peck, X. Wu, A. A. Tang, V. M. Wang, S. A. Bender, E. Lemire, R. Narayan, P. Montgomery, U. Ben-David, C. W. Garvie, Y. Chen, M. G. Rees, N. J. Lyons, J. M. McFarland, B. T. Wong, L. Wang, N. Dumont, P. J. O'Hearn, E. Stefan, J. G. Doench, C. N. Harrington, H. Greulich, M. Meyerson, F. Vazquez, A. Subramanian, J. A. Roth, J. A. Bittker, J. S. Boehm, C. C. Mader, A. Tsherniak, T. R.

Golub, Discovering the anti-cancer potential of non-oncology drugs by systematic viability profiling. *Nat. Cancer* **1**, 235–248 (2020).

22. G. Lukinavičius, K. Umezawa, N. Olivier, A. Honigmann, G. Yang, T. Plass, V. Mueller, L. Reymond, I. Corrêa, Z. Luo, C. Schultz, E. Lemke, P. Heppenstall, C. Eggeling, S. Manley, K. Johnsson, A near-infrared fluorophore for live-cell super-resolution microscopy of cellular proteins. *Nat. Chem.* **5**, 132–139 (2013).

23. M. Miller, R. Weissleder, Imaging of anticancer drug action in single cells. *Nat. Rev. Cancer* **17**, 399–414 (2017).

24. S. Regot, J. Hughey, B. Bajar, S. Carrasco, M. Covert, High-sensitivity measurements of multiple kinase activities in live single cells. *Cell* **157**, 1724–1734 (2014).

25. S. Lawrence, D. Nguyen, C. Bowen, L. Richards-Peterson, K. Skordos, The metabolic drug-drug interaction profile of dabrafenib: In vitro investigations and quantitative extrapolation of the P450-mediated DDI risk. *Drug Metab. Dispos.* **42**, 1180–1190 (2014).

26. A. King, M. Arnone, M. Bleam, K. Moss, J. Yang, K. Fedorowicz, K. Smitheman, J. Erhardt, A. Hughes-Earle, L. Kane-Carson, R. Sinnamon, H. Qi, T. Rheault, D. Uehling, S. Laquerre, Dabrafenib; preclinical characterization, increased efficacy when combined with trametinib, while BRAF/MEK tool combination reduced skin lesions. *PLOS ONE* **8**, e67583 (2013).

27. B. Homet Moreno, S. Mok, B. Comin-Anduix, S. Hu-Lieskovan, A. Ribas, Combined treatment with dabrafenib and trametinib with immune-stimulating antibodies for BRAF mutant melanoma. *Onco. Targets Ther.* **5**, e1052212 (2016).

28. K. T. Flaherty, J. R. Infante, A. Daud, R. Gonzalez, R. F. Kefford, J. Sosman, O. Hamid, L. Schuchter, J. Cebon, N. Ibrahim, R. Kudchadkar, H. A. Burris, G. Falchook, A. Algazi, K. Lewis, G. V. Long, I. Puzanov, P. Lebowitz, A. Singh, S. Little, P. Sun, A. Allred, D. Ouellet, K. B. Kim, K. Patel, J. Weber, Combined BRAF and MEK inhibition in melanoma with BRAF V600 mutations. *N. Engl. J. Med.* **367**, 1694–1703 (2012).

29. Y. Yoshii, K. Sugiyama, Intercapillary distance in the proliferating area of human glioma. *Cancer Res.* **48**, 2938–2941 (1988).

30. S. S. Basu, N. Y. R. Agar, Bringing matrix-assisted laser desorption/ionization mass spectrometry imaging to the clinics. *Clin. Lab. Med.* **41**, 309–324 (2021).

31. A. Loktev, T. Lindner, W. Mier, J. Debus, A. Altmann, D. Jäger, F. Giesel, C. Kratochwil, P. Barthe, C. Roumestand, U. Haberkorn, A tumor-imaging method targeting cancer-associated fibroblasts. *J. Nucl. Med.* **59**, 1423–1429 (2018).

32. D. J. Erstad, M. Sojoodi, M. S. Taylor, V. Clavijo Jordan, C. T. Farrar, A. L. Axtell, N. J. Rotile, C. Jones, K. A. Graham-O'Regan, D. S. Ferreira, T. Michelakos, F. Kontos, A. Chawla, S. Li, S. Ghoshal, Y. I. Chen, G. Arora, V. Humblet, V. Deshpande, M. Qadan, N. Bardeesy, C. R. Ferrone, M. Lanuti, K. K. Tanabe, P. Caravan, B. C. Fuchs, Fibrotic response to neoadjuvant therapy predicts survival in pancreatic cancer and is measurable with collagen-targeted molecular MRI. *Clin. Cancer Res.* **26**, 5007–5018 (2020).

33. N. I. Nissen, M. Karsdal, N. Willumsen, Collagens and cancer associated fibroblasts in the reactive stroma and its relation to cancer biology. *J. Exp. Clin. Cancer Res.* **38**, 115 (2019).

34. E. Sahai, I. Astsaturov, E. Cukierman, D. G. DeNardo, M. Egeblad, R. M. Evans, D. Fearon, F. R. Greten, S. R. Hingorani, T. Hunter, A framework for advancing our understanding of cancer-associated fibroblasts. *Nat. Rev. Cancer* **20**, 174–186 (2020).

35. C. D. Arvanitis, G. B. Ferraro, R. K. Jain, The blood–brain barrier and blood–tumour barrier in brain tumours and metastases. *Nat. Rev. Cancer* **20**, 26–41 (2020).

36. E. A. Wyatt, M. E. Davis, Method of establishing breast cancer brain metastases affects brain uptake and efficacy of targeted, therapeutic nanoparticles. *Bioeng. Transl. Med.* **4**, 30–37 (2019).

37. P. Becco, S. Gallo, S. Poletto, M. P. M. Frascione, L. Crotto, A. Zaccagna, L. Paruzzo, D. Caravelli, F. Carnevale-Schianca, M. Aglietta, Melanoma brain metastases in the era of target therapies: An overview. *Cancers (Basel)* **12**, 1640 (2020).

38. K. Holbrook, J. Lutzky, M. A. Davies, J. M. Davis, I. C. Glitza, R. N. Amaria, A. Diab, S. P. Patel, A. Amin, H. Tawbi, Intracranial antitumor activity with encorafenib plus binimetinib in patients with melanoma brain metastases: A case series. *Cancer* **126**, 523–530 (2020).

39. D. R. Hargrave, E. Bouffet, U. Tabori, A. Broniscer, K. J. Cohen, J. R. Hansford, B. Geoerger, P. Hingorani, I. J. Dunkel, M. W. Russo, L. Tseng, K. Dasgupta, E. Gasal, J. A. Whitlock, M. W. Kieran, Efficacy and safety of dabrafenib in pediatric patients with *BRAF* V600 mutation–positive relapsed or refractory low-grade glioma: Results from a phase I/IIa study. *Clin. Cancer Res.* **25**, 7303–7311 (2019).

40. G. Falchook, G. Long, R. Kurzrock, K. Kim, H. Arkenau, M. Brown, O. Hamid, J. Infante, M. Millward, A. Pavlick, M. Chin, S. O'Day, S. Blackman, C. Curtis, P. Lebowitz, B. Ma, D. Ouellet, R. Kefford, Dose selection, pharmacokinetics, and pharmacodynamics of BRAF inhibitor dabrafenib (GSK2118436). *Clin. Cancer Res.* **20**, 4449–4458 (2014).

41. L. Gerosa, C. Chidley, F. Fröhlich, G. Sanchez, S. K. Lim, J. Muhlich, J.-Y. Chen, S. Vallabhaneni, G. J. Baker, D. Schapiro, M. I. Atanasova, L. A. Chylek, T. Shi, L. Yi, C. D. Nicora, A. Claas, T. S. C. Ng, R. H. Kohler, D. A. Lauffenburger, R. Weissleder, M. A. Miller, W.-J. Qian, H. S. Wiley, P. K. Sorger, Receptor-driven ERK pulses reconfigure MAPK signaling and enable persistence of drug-adapted BRAF-mutant melanoma cells. *Cell Syst.* **11**, 478–494.e9 (2020).

42. A. Palmer, P. Sorger, Combination cancer therapy can confer benefit via patient-to-patient variability without drug additivity or synergy. *Cell* **171**, 1678–1691.e13 (2017).

43. L. Heinzerling, T. K. Eigentler, M. Fluck, J. C. Hassel, D. Heller-Schenck, J. Leipe, M. Pauschinger, A. Vogel, L. Zimmer, R. Gutzmer, Tolerability of BRAF/MEK inhibitor combinations: Adverse event evaluation and management. *ESMO Open* **4**, e000491 (2019).

44. Z. Suo, X. Xiong, Q. Sun, L. Zhao, P. Tang, Q. Hou, Y. Zhang, D. Wu, H. Li, Investigation on the interaction of dabrafenib with human serum albumin using combined experiment and molecular dynamics simulation: Exploring the binding mechanism, esterase-like activity, and antioxidant activity. *Mol. Pharm.* **15**, 5637–5645 (2018).

45. D. J. Adams, L. R. Morgan, Tumor physiology and charge dynamics of anticancer drugs: Implications for camptothecin-based drug development. *Curr. Med. Chem.* **18**, 1367–1372 (2011).

46. R. Barnhill, P.-J. van Dam, P. Vermeulen, G. Champenois, A. Nicolas, R. V. Rawson, J. S. Wilmott, J. F. Thompson, G. V. Long, N. Cassoux, S. Roman-Roman, K. J. Busam, R. A. Scolyer, A. J. Lazar, C. Lugassy, Replacement and desmoplastic histopathological growth patterns in cutaneous melanoma liver metastases: Frequency, characteristics, and robust prognostic value. *J. Pathol. Clin. Res.* **6**, 195–206 (2020).

47. K. Nielsen, H. C. Rolff, R. L. Eefsen, B. Vainer, The morphological growth patterns of colorectal liver metastases are prognostic for overall survival. *Mod. Pathol.* **27**, 1641–1648 (2014).

48. B. Galjart, P. M. H. Nierop, E. P. van der Stok, R. R. J. C. van den Braak, D. J. Höppener, S. Daelemans, L. Y. Dirix, C. Verhoef, P. B. Vermeulen, D. J. Grünhagen, Angiogenic desmoplastic histopathological growth pattern as a prognostic marker of good outcome in patients with colorectal liver metastases. *Angiogenesis* **22**, 355–368 (2019).

49. S. Frentzas, E. Simoneau, V. L. Bridgeman, P. B. Vermeulen, S. Foo, E. Kostaras, M. Nathan, A. Wotherspoon, Z.-H. Gao, Y. Shi, G. Van den Eynden, F. Daley, C. Peckitt, X. Tan, A. Salman, A. Lazaris, P. Gazinska, T. J. Berg, Z. Eltahir, L. Ritsma, J. Van Rheenen, A. Khashper, G. Brown, H. Nystrom, M. Sund, S. Van Laere, E. Loyer, L. Dirix, D. Cunningham, P. Metrakos, A. R. Reynolds, Vessel co-option mediates resistance to anti-angiogenic therapy in liver metastases. *Nat. Med.* **22**, 1294–1302 (2016).

50. T. S. C. Ng, R. T. Seethamraju, R. Bueno, R. R. Gill, Clinical implementation of a free-breathing, motion-robust dynamic contrast-enhanced mri protocol to evaluate pleural tumors. *AJR Am. J. Roentgenol.* **215**, 94–104 (2020).

51. S. R. Barnes, T. S. Ng, A. Montagne, M. Law, B. V. Zlokovic, R. E. Jacobs, Optimal acquisition and modeling parameters for accurate assessment of low Ktrans blood-brain barrier permeability using dynamic contrast-enhanced MRI. *Magn. Reson. Med.* **75**, 1967–1977 (2016).

52. R. Li, T. S. C. Ng, S. J. Wang, M. Prytyskach, C. B. Rodell, H. Mikula, R. H. Kohler, M. A. Garlin, D. A. Lauffenburger, S. Parangi, D. M. Dinulescu, N. Bardeesy, R. Weissleder, M. A. Miller, Therapeutically reprogrammed nutrient signalling enhances nanoparticulate albumin bound drug uptake and efficacy in KRAS-mutant cancer. *Nat. Nanotechnol.* **16**, 830–839 (2021).

53. B. Finicle, V. Jayashankar, A. Edinger, Nutrient scavenging in cancer. *Nat. Rev. Cancer* **18**, 619–633 (2018).

54. D. Liston, M. Davis, Clinically relevant concentrations of anticancer drugs: A guide for nonclinical studies. *Clin. Cancer Res.* **23**, 3489–3498 (2017).

55. A. Zorzi, S. Linciano, A. Angelini, Non-covalent albumin-binding ligands for extending the circulating half-life of small biotherapeutics. *MedChemComm* **10**, 1068–1081 (2019).

56. J. Wu, P. M. Lorusso, L. H. Matherly, J. Li, Implications of plasma protein binding for pharmacokinetics and pharmacodynamics of the γ-secretase inhibitor RO4929097. *Clin. Cancer Res.* **18**, 2066–2079 (2012).

57. D. A. Smith, L. Di, E. H. Kerns, The effect of plasma protein binding on in vivo efficacy: Misconceptions in drug discovery. *Nat. Rev. Drug Discov.* **9**, 929–939 (2010).

58. J. Zhang, L. Lang, Z. Zhu, F. Li, G. Niu, X. Chen, Clinical translation of an albumin-binding PET radiotracer 68Ga-NEB. *J. Nucl. Med.* **56**, 1609–1614 (2015).

59. Z. Liu, X. Chen, Simple bioconjugate chemistry serves great clinical advances: Albumin as a versatile platform for diagnosis and precision therapy. *Chem. Soc. Rev.* **45**, 1432–1456 (2016).

60. P. Bannas, C. A. Bookwalter, T. Ziemlewicz, U. Motosugi, A. M. Del Rio, T. A. Potretzke, S. K. Nagle, S. B. Reeder, Combined gadoxetic acid and gadofosveset enhanced liver MRI for detection and characterization of liver metastases. *Eur. Radiol.* **27**, 32–40 (2017).

61. J. Puig, G. Blasco, J. Daunis-i-Estadella, A. Alberich-Bayarri, M. Essig, R. Jain, S. Remollo, D. Hernández, M. Puigdemont, J. Sánchez-González, High-resolution blood-pool-contrast-enhanced MR

angiography in glioblastoma: Tumor-associated neovascularization as a biomarker for patient survival. A preliminary study. *Neuroradiology* **58**, 17–26 (2016).

62. J. Do, D. Foster, C. Renier, H. Vogel, S. Rosenblum, T. C. Doyle, V. Tse, I. Wapnir, Ex vivo Evans blue assessment of the blood brain barrier in three breast cancer brain metastasis models. *Breast Cancer Res. Treat.* **144**, 93–101 (2014).

63. T. Lin, P. Zhao, Y. Jiang, Y. Tang, H. Jin, Z. Pan, H. He, V. C. Yang, Y. Huang, Blood–brain-barrier-penetrating albumin nanoparticles for biomimetic drug delivery via albumin-binding protein pathways for antiglioma therapy. *ACS Nano* **10**, 9999–10012 (2016).

64. M. H. Dornan, D. Petrenyov, J.-M. Simard, M. Boudjemeline, R. Mititelu, J. N. DaSilva, A. P. Belanger, Synthesis of a ^{11}C-isotopologue of the B-Raf-selective inhibitor encorafenib using in-loop [^{11}C]CO_2 fixation. *ACS Omega* **5**, 20960–20966 (2020).

65. E. C. Pratt, E. Isaac, E. P. Stater, G. Yang, O. Ouerfelli, N. Pillarsetty, J. Grimm, Synthesis of the PET tracer ^{124}I-trametinib for MAPK/ERK kinase distribution and resistance monitoring. *J. Nucl. Med.* **61**, 1845–1850 (2020).

66. C. Jiang, L. Xie, Y. Zhang, M. Fujinaga, W. Mori, Y. Kurihara, T. Yamasaki, F. Wang, M.-R. Zhang, Pharmacokinetic evaluation of [^{11}C]CEP-32496 in nude mice bearing $BRAF^{V600E}$ mutation-induced melanomas. *Mol. Imaging* **17**, 1536012118795952 (2018).

67. M. L. Maitland, R. E. Cearbhaill, J. Gobburu, Cancer clinical investigators should converge with pharmacometricians. *Clin. Cancer Res.* **25**, 5182–5184 (2019).

68. T. S. C. Ng, M. A. Garlin, R. Weissleder, M. A. Miller, Improving nanotherapy delivery and action through image-guided systems pharmacology. *Theranostics* **10**, 968–997 (2020).

69. S. Wang, R. Li, T. Ng, G. Luthria, M. Oudin, M. Prytyskach, R. Kohler, R. Weissleder, D. Lauffenburger, M. Miller, Efficient blockade of locally reciprocated tumor-macrophage signaling using a TAM-avid nanotherapy. *Sci. Adv.* **6**, eaaz8521 (2020).

70. J. Schindelin, I. Arganda-Carreras, E. Frise, V. Kaynig, M. Longair, T. Pietzsch, S. Preibisch, C. Rueden, S. Saalfeld, B. Schmid, J. Tinevez, D. White, V. Hartenstein, K. Eliceiri, P. Tomancak, A. Cardona, Fiji: An open-source platform for biological-image analysis. *Nat. Methods* **9**, 676–682 (2012).

71. C. McQuin, A. Goodman, V. Chernyshev, L. Kamentsky, B. Cimini, K. Karhohs, M. Doan, L. Ding, S. Rafelski, D. Thirstrup, W. Wiegraebe, S. Singh, T. Becker, J. Caicedo, A. Carpenter, CellProfiler 3.0: Next-generation image processing for biology. *PLOS Biol.* **16**, e2005970 (2018).

72. R. Lowry, Concepts and applications of inferential statistics (2014); *doer.col.org*.

73. M. Dreher, W. Liu, C. Michelich, M. Dewhirst, F. Yuan, A. Chilkoti, Tumor vascular permeability, accumulation, and penetration of macromolecular drug carriers. *J. Natl. Cancer Inst.* **98**, 335–344 (2006).

74. L. Gerosa, C. Chidley, F. Froehlich, G. Sanchez, S. Lim, J. Muhlich, J. Chen, G. Baker, D. Schapiro, T. Shi, Sporadic ERK pulses drive non-genetic resistance in drug-adapted $BRAF^{V600E}$ melanoma cells. *Cell Syst.* **11**, 478–494.e9 (2020).

75. A. Van der Veldt, M. Lubberink, I. Bahce, M. Walraven, M. de Boer, H. Greuter, N. Hendrikse, J. Eriksson, A. Windhorst, P. Postmus, H. Verheul, E. Serné, A. Lammertsma, E. Smit, Rapid decrease in delivery of chemotherapy to tumors after anti-VEGF therapy: Implications for scheduling of anti-angiogenic drugs. *Cancer Cell* **21**, 82–91 (2012).

76. J. Wang, C. Perry, K. Meeth, D. Thakral, W. Damsky, G. Micevic, S. Kaech, K. Blenman, M. Bosenberg, UV-induced somatic mutations elicit a functional T cell response in the YUMMER1.7 mouse melanoma model. *Pigment Cell Melanoma Res.* **30**, 428–435 (2017).

77. J. Tate, S. Bamford, H. Jubb, Z. Sondka, D. Beare, N. Bindal, H. Boutselakis, C. Cole, C. Creatore, E. Dawson, P. Fish, B. Harsha, C. Hathaway, S. Jupe, C. Kok, K. Noble, L. Ponting, C. Ramshaw, C. Rye, H. Speedy, R. Stefancsik, S. Thompson, S. Wang, S. Ward, P. Campbell, S. Forbes, COSMIC: The catalogue of somatic mutations in cancer. *Nucleic Acids Res.* **47**, D941–D947 (2019).

78. D. Pépin, A. Sosulski, L. Zhang, D. Wang, V. Vathipadiekal, K. Hendren, C. Coletti, A. Yu, C. Castro, M. Birrer, G. Gao, P. Donahoe, AAV9 delivering a modified human Mullerian inhibiting substance as a gene therapy in patient-derived xenografts of ovarian cancer. *Proc. Natl. Acad. Sci. U.S.A.* **112**, E4418–E4427 (2015).

79. A. Laughney, E. Kim, M. Sprachman, M. Miller, R. Kohler, K. Yang, J. Orth, T. Mitchison, R. Weissleder, Single-cell pharmacokinetic imaging reveals a therapeutic strategy to overcome drug resistance to the microtubule inhibitor eribulin. *Sci. Transl. Med.* **6**, 261ra152 (2014).

80. M. Fallahi-Sichani, V. Becker, B. Izar, G. Baker, J. Lin, S. Boswell, P. Shah, A. Rotem, L. Garraway, P. Sorger, Adaptive resistance of melanoma cells to RAF inhibition via reversible induction of a slowly dividing de-differentiated state. *Mol. Syst. Biol.* **13**, 905 (2017).

81. J. Sarkaria, D. Ma, M. Schroeder, B. Carlson, C. Giannini, I. Parney, PM-19: Development of a panel of patient-derived xenograft (PDX) models from brain metastases. *Neuro Oncol.* **16**, v173 (2014).

82. Y. Wang, S. Liu, Z. Yang, A. P. Algazi, S. H. Lomeli, Y. Wang, M. Othus, A. Hong, X. Wang, C. E. Randolph, A. M. Jones, M. W. Bosenberg, S. D. Byrum, A. J. Tackett, H. Lopez, C. Yates, D. B. Solit, A. Ribas, M. Piva, G. Moriceau, R. S. Lo, Anti-PD-1/L1 lead-in before MAPK inhibitor combination maximizes antitumor immunity and efficacy. *Cancer Cell* **39**, 1375–1387.e6 (2021).

83. M. R. Duncan, K. S. Frazier, S. Abramson, S. Williams, H. Klapper, X. Huang, G. R. Grotendorst, Connective tissue growth factor mediates transforming growth factor β-induced collagen synthesis: Down-regulation by cAMP. *FASEB J.* **13**, 1774–1786 (1999).

84. A. López-De León, M. Rojkind, A simple micromethod for collagen and total protein determination in formalin-fixed paraffin-embedded sections. *J. Histochem. Cytochem.* **33**, 737–743 (1985).

85. M. N. O. Sadiku, C. N. Obiozor, A simple introduction to the method of lines. *Int. J. Electr. Eng. Educ.* **37**, 282–296 (2000).

86. I. Waizenegger, A. Baum, S. Steurer, H. Stadtmüller, G. Bader, O. Schaaf, P. Garin-Chesa, A. Schlattl, N. Schweifer, C. Haslinger, F. Colbatzky, S. Mousa, A. Kalkuhl, N. Kraut, G. Adolf, A

novel RAF kinase inhibitor with DFG-out-binding mode: High efficacy in braf-mutant tumor xenograft models in the absence of normal tissue hyperproliferation. *Mol. Cancer Ther.* **15**, 354–365 (2016).

87. C. Kuhl, RECIST needs revision: A wake-up call for radiologists. *Radiology* **292**, 110–111 (2019).

88. S. Vaidhyanathan, B. Wilken-Resman, D. J. Ma, K. E. Parrish, R. K. Mittapalli, B. L. Carlson, J. N. Sarkaria, W. F. Elmquist, Factors influencing the central nervous system distribution of a novel phosphoinositide 3-kinase/mammalian target of rapamycin inhibitor GSK2126458: Implications for overcoming resistance with combination therapy for melanoma brain metastases. *J. Pharmacol. Exp. Ther.* **356**, 251–259 (2016).

89. M. A. Miller, R. Chandra, M. F. Cuccarese, C. Pfirschke, C. Engblom, S. Stapleton, U. Adhikary, R. H. Kohler, J. F. Mohan, M. J. Pittet, R. Weissleder, Radiation therapy primes tumors for nanotherapeutic delivery via macrophage-mediated vascular bursts. *Sci. Transl. Med.* **9**, eaal0225 (2017).

90. T. R. Rheault, J. C. Stellwagen, G. M. Adjabeng, K. R. Hornberger, K. G. Petrov, A. G. Waterson, S. H. Dickerson, R. A. Mook, Jr., S. G. Laquerre, A. J. King, O. W. Rossanese, M. R. Arnone, K. N. Smitheman, L. S. Kane-Carson, C. Han, G. S. Moorthy, K. G. Moss, D. E. Uehling, Discovery of dabrafenib: A selective inhibitor of raf kinases with antitumor activity against b-raf-driven tumors. *ACS Med. Chem. Lett.* **4**, 358–362 (2013).

91. R. J. Sullivan, J. Weber, S. Patel, R. Dummer, M. S. Carlino, D. S. Tan, C. Lebbé, S. Siena, E. Elez, L. Wollenberg, A phase Ib/II study of the BRAF inhibitor encorafenib plus the MEK inhibitor binimetinib in patients with BRAFV600E/K-mutant solid tumors. *Clin. Cancer Res.* **26**, 5102–5112 (2020).

92. D. B. Johnson, K. T. Flaherty, J. S. Weber, J. R. Infante, K. B. Kim, R. F. Kefford, O. Hamid, L. Schuchter, J. Cebon, W. H. Sharfman, R. R. McWilliams, M. Sznol, D. P. Lawrence, G. T. Gibney, H. A. Burris III, G. S. Falchook, A. Algazi, K. Lewis, G. V. Long, K. Patel, N. Ibrahim, P. Sun, S. Little, E. Cunningham, J. A. Sosman, A. Daud, R. Gonzalez, Combined BRAF (dabrafenib) and

MEK inhibition (trametinib) in patients with BRAFV600-mutant melanoma experiencing progression with single-agent BRAF inhibitor. *J. Clin. Oncol.* **32**, 3697–3704 (2014).

93. G. M. Thurber, K. S. Yang, T. Reiner, R. H. Kohler, P. Sorger, T. Mitchison, R. Weissleder, Single-cell and subcellular pharmacokinetic imaging allows insight into drug action in vivo. *Nat. Commun.* **4**, 1504 (2013).

94. G. M. Thurber, R. Weissleder, A systems approach for tumor pharmacokinetics. *PLOS ONE* **6**, e24696 (2011).

95. L. J. Nugent, R. K. Jain, Extravascular diffusion in normal and neoplastic tissues. *Cancer Res.* **44**, 238–244 (1984).

96. K. Atkovska, J. Klingler, J. Oberwinkler, S. Keller, J. S. Hub, Rationalizing steroid interactions with lipid membranes: Conformations, partitioning, and kinetics. *ACS Cent. Sci.* **4**, 1155–1165 (2018).

97. F. Yuan, M. Dellian, D. Fukumura, M. Leunig, D. A. Berk, V. P. Torchilin, R. K. Jain, Vascular permeability in a human tumor xenograft: Molecular size dependence and cutoff size. *Cancer Res.* **55**, 3752–3756 (1995).

98. F. Yuan, M. Leunig, D. A. Berk, R. K. Jain, Microvascular permeability of albumin, vascular surface area, and vascular volume measured in human adenocarcinoma LS174T using dorsal chamber in SCID mice. *Microvasc. Res.* **45**, 269–289 (1993).

99. F. Yuan, M. Leunig, S. K. Huang, D. A. Berk, D. Papahadjopoulos, R. K. Jain, Mirovascular permeability and interstitial penetration of sterically stabilized (stealth) liposomes in a human tumor xenograft. *Cancer Res.* **54**, 3352–3356 (1994).

100. S. M. Davidson, O. Jonas, M. A. Keibler, H. W. Hou, A. Luengo, J. R. Mayers, J. Wyckoff, A. M. Del Rosario, M. Whitman, C. R. Chin, Direct evidence for cancer-cell-autonomous extracellular protein catabolism in pancreatic tumors. *Nat. Med.* **23**, 235–241 (2017).

101. T. D. Williams, R. R. Kay, The physiological regulation of macropinocytosis during Dictyostelium growth and development. *J. Cell Sci.* **131**, jcs213736 (2018).

102. F. Sallusto, M. Cella, C. Danieli, A. Lanzavecchia, Dendritic cells use macropinocytosis and the mannose receptor to concentrate macromolecules in the major histocompatibility complex class II compartment: Downregulation by cytokines and bacterial products. *J. Exp. Med.* **182**, 389–400 (1995).

103. M. Amyere, B. Payrastre, U. Krause, P. V. D. Smissen, A. Veithen, P. J. Courtoy, Constitutive macropinocytosis in oncogene-transformed fibroblasts depends on sequential permanent activation of phosphoinositide 3-kinase and phospholipase C. *Mol. Biol. Cell* **11**, 3453–3467 (2000).

104. K. Holt, M. Ye, S. Nagar, K. Korzekwa, Prediction of tissue-plasma partition coefficients using microsomal partitioning: Incorporation into physiologically based pharmacokinetic models and steady-state volume of distribution predictions. *Drug Metab. Dispos.* **47**, 1050–1060 (2019).

105. J. Portoukalian, G. Zwingelstein, J.-F. DORÉ, Lipid composition of human malignant melanoma tumors at various levels of malignant growth. *Eur. J. Biochem.* **94**, 19–23 (1979).

106. L. Adlung, S. Kar, M. C. Wagner, B. She, S. Chakraborty, J. Bao, S. Lattermann, M. Boerries, H. Busch, P. Wuchter, Protein abundance of AKT and ERK pathway components governs cell type-specific regulation of proliferation. *Mol. Syst. Biol.* **13**, 904 (2017).

107. M. A. Miller, B. Askevold, K. S. Yang, R. H. Kohler, R. Weissleder, Platinum compounds for high-resolution in vivo cancer imaging. *ChemMedChem* **9**, 1131–1135 (2014).

108. A. G. Gilmartin, M. R. Bleam, A. Groy, K. G. Moss, E. A. Minthorn, S. G. Kulkarni, C. M. Rominger, S. Erskine, K. E. Fisher, J. Yang, GSK1120212 (JTP-74057) is an inhibitor of MEK activity and activation with favorable pharmacokinetic properties for sustained in vivo pathway inhibition. *Clin. Cancer Res.* **17**, 989–1000 (2011).

109. Z. M. Khan, A. M. Real, W. M. Marsiglia, A. Chow, M. E. Duffy, J. R. Yerabolu, A. P. Scopton, A. C. Dar, Structural basis for the action of the drug trametinib at KSR-bound MEK. *Nature* **588**, 509–514 (2020).

110. S. Modok, P. Hyde, H. R. Mellor, T. Roose, R. Callaghan, Diffusivity and distribution of vinblastine in three-dimensional tumour tissue: Experimental and mathematical modelling. *Eur. J. Cancer* **42**, 2404–2413 (2006).
